# Molecular Signature in Focal Cortical Dysplasia: A Systematic Review of RNA and Protein Data

**DOI:** 10.3390/ijms26209909

**Published:** 2025-10-11

**Authors:** Jalleh Shakerzadeh, Radim Jaroušek, Zita Goliášová, Milan Brázdil

**Affiliations:** 1 Brno Epilepsy Centre, 1st Department of Neurology, St. Anne’s University Hospital, Faculty of Medicine, Masaryk University, 602 00 Brno, Czech Republic; jalleh.shakerzadeh@med.muni.cz (J.S.); radim.jarousek@med.muni.cz (R.J.); z.goliasova@gmail.com (Z.G.); 2 Department of Experimental Biology, Faculty of Science, Masaryk University, Kotlářská 2, 602 00 Brno, Czech Republic; 3 Behavioral and Social Neuroscience Research Group, CEITEC, Masaryk University, 625 00 Brno, Czech Republic

**Keywords:** focal cortical dysplasia, drug-resistant epilepsy, molecular biomarkers, neuroinflammation, synaptic plasticity, transcriptome, proteome, microRNAs, pathway analysis

## Abstract

Focal cortical dysplasia (FCD) is a major cause of drug-resistant epilepsy, yet its molecular basis remains poorly understood. Numerous studies have analyzed RNA, protein, and microRNA alterations, but results are often inconsistent across subtypes and methodologies. To address this gap, we conducted a systematic review integrating transcriptomic, proteomic, and microRNA data from 117 human studies of FCD subtypes I–III. Differentially expressed factors were extracted, categorized by subtype, and analyzed using pathway enrichment and network approaches. Our integrative analysis revealed convergent dysregulation of neuroinflammatory, synaptic, cytoskeletal, and metabolic pathways across FCD subtypes. Consistently altered genes, including IL1B, TLR4, BDNF, HMGCR, and ROCK2, together with dysregulated microRNAs such as hsa-miR-21-5p, hsa-miR-155-5p, and hsa-miR-132-3p, were linked to PI3K–Akt–mTOR, Toll-like receptor, and GABAergic signaling, emphasizing shared pathogenic mechanisms. Importantly, we identified overlapping transcript–protein patterns and subtype-specific molecular profiles that may refine diagnosis and inform therapeutic strategies. This review provides the first cross-omics molecular framework of FCD, demonstrating how convergent pathways unify heterogeneous findings and offering a roadmap for biomarker discovery and targeted interventions.

## 1. Introduction

Focal cortical dysplasia (FCD) is an organizational and developmental malformation of cortical neurons. It is often a leading cause of drug-resistant epilepsy, especially in pediatric patients. Disease identification has evolved over the past 50 years [1], and the complete consensus classification of several histopathological features led to the establishment of categories by the International League Against Epilepsy (ILAE), which are divided into three types [2], with the last update in 2022 [3]. FCD is categorized through the recognition of cortical dyslamination (type I), which includes radial (Ia), tangential (Ib), or a combination of both (Ic). Type II is characterized by cortical dyslamination along with dysmorphic neurons, either without balloon cells (IIa) or with balloon cells (IIb), and type III is associated with other principal lesions, such as hippocampal sclerosis or tumors [3].

Despite progress in histopathological and high-resolution imaging, such as 7 Tesla MRI [4], the diagnosis of FCD remains challenging due to the subtlety and heterogeneity of lesions. Unsuccessful medical treatment of FCD leads to surgical intervention, with a success rate of approximately 50% to 55% [5]; however, postoperative outcomes vary, and permanent disability can persist even after seizure control is achieved [6]. An ideal approach would integrate molecular data with traditional diagnostic tools to enhance precision and improve treatment outcomes.

FCD is believed to arise from abnormalities in neuronal migration during brain development, involving both somatic and germline mutations [7]. These genetic and epigenetic disturbances result in a wide range of molecular and cellular alterations, underscoring the potential for identifying diagnostic biomarkers and therapeutic targets.

What is currently lacking in the field is a comprehensive approach that integrates analysis and compares data across subtypes and omics layers. The majority of prior reviews focused on one omics layer (transcriptomics or proteomics) or provided narrative overviews without integrating expression evidence across data types. Here, we conduct a systematic, cross-omics analysis of 117 human studies that integrates miRNA, mRNA, and protein data in focal cortical dysplasia. To assess consistency across layers, we map miRNAs to their reported mRNA targets and proteins, as well as harmonize ILAE subtypes (I, IIa/IIb, III). The aggregation of results at the pathway/network level reveals convergent mechanisms, including innate immune activation, chloride homeostasis/GABArgic tone, cytoskeletal/ECM remodeling, and cholesterol/mevalonate metabolism, not evident in single-layer summaries. A cross-layer, subtype-aware framework explains much of the apparent heterogeneity and provides reproducible guidelines for biomarker discovery, target prioritization, and study design.

## 2. Materials and Methods

This review was registered with PROSPERO (registration number: CRD42024611156), and the protocol is available at http://www.crd.york.ac.uk/PROSPERO (accessed on 6 October 2025).

### 2.1. Search Design

**Figure 1:**
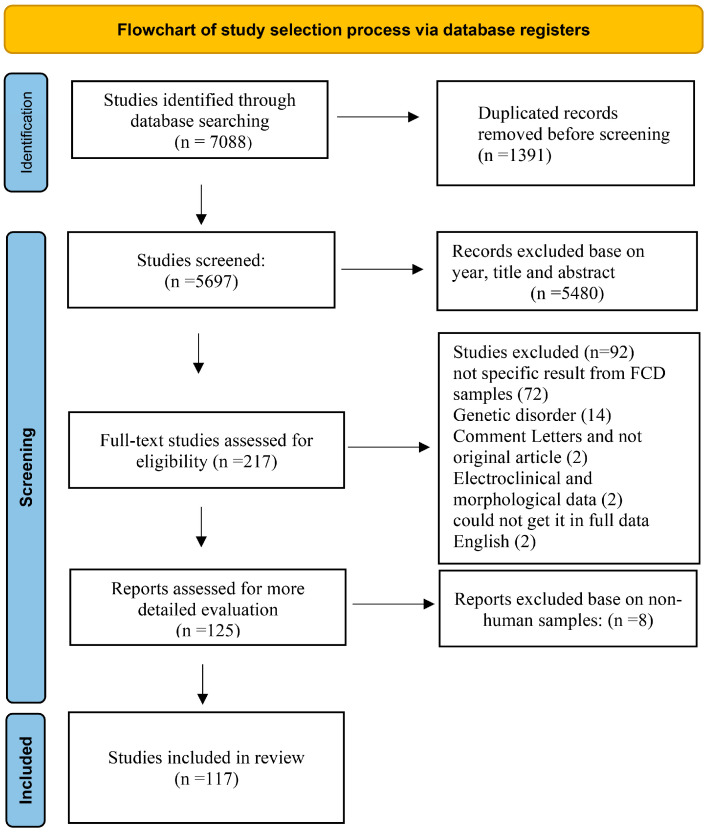
PRISMA flow diagram following the PRISMA checklist for study selection.

This systematic review was conducted in accordance with the PRISMA 2020 guidelines. A completed PRISMA 2020 checklist is provided as Supplementary File S1, and the study selection process is summarized in a PRISMA flow diagram (Figure 1). A literature search was conducted using PubMed, Web of Science, Scopus, and Science Direct for investigating differentially expressed miRNAs, mRNAs, and protein data. Search items included: (Focal Cortical Dysplasia OR FCD OR Cortical Dysplasia) AND (miRNA OR microRNA OR miR), (Focal Cortical Dysplasia OR FCD OR Cortical Dysplasia) AND (Transcriptomics OR Microarray OR RNA-sequencing), (Focal Cortical Dysplasia OR FCD OR Cortical dysplasia) AND (Proteomics OR protein profiling OR Mass spectrometry) AND (Focal Cortical Dysplasia OR FCD OR Cortical dysplasia) AND (protein expression OR protein analysis OR Western blot OR ELISA OR immunohistochemistry OR IHC OR immunostaining). Reference lists of relevant articles were also screened manually to identify additional eligible studies. The complete screening process is illustrated in Figure 1.

### 2.2. Eligibility Criteria

Studies were included if they met the following criteria: patients diagnosed with FCD. Case-control studies focus on FCD and provide RNA, protein, or microRNA data related to FCD. Studies using microarrays, Western blotting (WB), immunohistochemistry (IHC), and proteomics for molecular profiling are among the most common and were published from 2004 to August 2024. Excluded studies were those where (1) FCD derives from genetic mutations; (2) data are incomplete; (3) data are duplicated; (4) there are review and theoretical articles, letters, or conferences; (5) there are nonhuman samples; and (6) they are not published in English.

### 2.3. Study Selection Workflow

To ensure reproducibility and transparency, the study selection process followed a structured, multi-step screening strategy based on PRISMA guidelines.

Database Search and Record Collection:

Literature searches were conducted in PubMed, Web of Science, Scopus, and Science Direct using predefined keyword combinations (see Section 2.1). A total of 7088 records were retrieved across all databases. Additionally, reference lists of relevant articles were manually screened to identify further eligible studies. All records were exported into Microsoft Excel, and duplicate entries were removed using both automated filters and manual inspection. After deduplication, 5697 unique records remained for screening. For title and abstract Screening, two reviewers independently screened titles and abstracts for relevance. Articles were excluded if they were (1) published before 2004, (2) non-English language articles, or (3) unrelated to focal cortical dysplasia or lacked molecular analysis. As a result, 5480 records were excluded at this stage. Discrepancies between reviewers were resolved through discussion.

Full-text Screening: The remaining 217 full-text articles were independently reviewed by the same two reviewers. The inclusion and exclusion criteria (see Section 2.2) were applied in detail. Studies were excluded for the following reasons: non-human models (*n* = 8), genetic mutation studies rather than transcript/protein expression (*n* = 14), incomplete, unclear, or non-specific molecular data (*n* = 72), morphological data only (*n* = 2), inaccessible full text in English (*n* = 2), and comment letters (*n* = 2).

Final Inclusion and Data Extraction:

A total of 117 articles met the eligibility criteria and were included in the final review. These comprised 8 studies on microRNAs, 28 studies on mRNA expression, and 98 studies on protein-level changes (some studies contributed to more than one category). All steps of study selection and exclusion were performed using a reproducible and transparent workflow, as outlined above and visualized in the PRISMA diagram (Figure 1).

### 2.4. Data Extraction

Data extraction was carefully screened and recovered by two writers working independently. The final process of selection was discussed with a third author regarding any discrepancies until an agreement was reached. For every study included, the following data were taken: the first author, the FCD type, the specimen type, the case and control number, the age, the methodology, and so on.

### 2.5. Statistical and Bioinformatics Analysis

All data processing and visualization were performed in the R programming language via the dplyR (version 1.1.4) [8] and tidyR (version 1.3.1) packages [9]. The workflow scheme is available in Supplementary File S2.

Data standardization: Gene and protein identifiers were standardized using HGNC (2025-01-08) [10], UniProtKB (version 2025-01) [11], and GeneCards (version 5.25.0) [12], with final validation via the gprofiler2 package (ver. 0.2.3) (R) [13]. Additionally, miRNA names were standardized to the hsa-miR-number format via the MultiMiR package (version 1.30) (R) [14]. For network visualization, we constructed a layered bipartite network to integrate literature references, overlapping proteins/genes, and enriched biological pathways. Pathway enrichment was performed using the clusterProfiler (version 4.16.0) package with the GO Biological Process (GO_BP) database. References were placed in the top layer, with edges connecting them to proteins/genes based on reported associations in published studies. Proteins/genes (colored according to up- or down-regulation) were positioned in the middle layer, while enriched pathways were displayed in the bottom layer. The network was generated in R using the tidygraph (version 1.3.1) and ggraph (version 2.0) packages, with manual layout adjustments to ensure clear layer separation and pastel color coding for visual interpretability. Supporting tables were prepared to provide the list of underlying publications and detailed descriptions of the pathways included in the network.

Dysregulation and Overlap Analysis: Identified genes and proteins were categorized into two categories: unique (one study only) and overlapping. Nested bar plots were used to visualize recurrence and direction of regulation.

miRNA–mRNA–protein interaction: Overlapping genes and proteins were matched and cross-referenced with validated miRNA targets using the MultiMiR (version 1.30) package. Functional enrichment was performed using the EnrichR (version 3.4) and gprofiler2 (version 0.2.3) packages [15] referencing WikiPathways. Visualizations were generated using ggplot2 (version 3.5.2) [16].

Data Visualization: Circular visualizations of associations between dysregulated molecules and FCD subtypes were generated using the Circos package (version 0.4.15) [17].

Interactive tables: The tables were created by the DT package (version 0.34) (R) in html format. Data could be downloaded directly from the html file in various formats (Excel, csv, and PDF). The interactive tables were tested in the latest versions of Mozilla Firefox, Google Chrome, and Microsoft Edge.

Data availability: Data could be accessed through https://doi.org/10.5281/zenodo.15786178 (accessed on 1 October 2025) and R scripts are available on demand. No formal risk-of-bias tool was applied because of the heterogeneity of study designs and methodologies. Instead, potential sources of bias (differences in classification systems, patient demographics, tissue quality, and analytical platforms) were qualitatively evaluated and are discussed in Section State of the Field and Future Research and Recommendations. Extracted measures included fold change, log2 fold change, and *p*-values when available; presence/absence data from IHC/WB were recorded qualitatively. No pooled quantitative effect sizes were calculated; results were synthesized descriptively and stratified by FCD subtype. No sensitivity analyses were conducted. Certainty of evidence was inferred from the consistency of findings across multiple independent studies and across omics layers, with findings replicated in at least two independent cohorts or confirmed at both transcriptomic and proteomic levels considered higher confidence.

## 3. Results

### 3.1. Overview of Reviewed Studies Across FCD Subtypes

**Figure 2:**
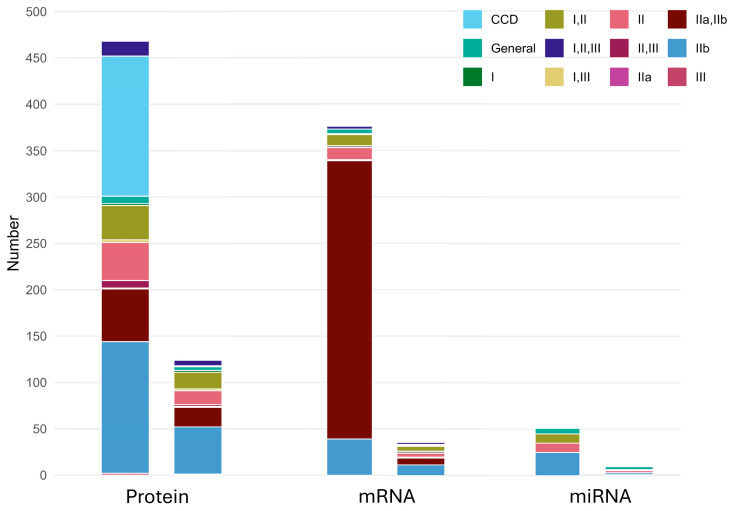
Side-by-side stacked bar plots summarizing FCD coverage. The left bar depicts the number of molecules reported per FCD type, and the right bar the number of publications per FCD type. Colors indicate FCD types (see Legend). In our review, protein evidence is dominated by CCD (Childhood Cortical Dysplasia) and IIb, whereas the majority of mRNA data belongs to type IIa/IIb. A limited number of studies on miRNA dysregulation were obtained, with a focus mostly on type IIb.

To contextualize the molecular landscape analyzed in this review, we first mapped the distribution of available studies and reported molecules across focal cortical dysplasia (FCD) subtypes (Figure 2). Most of the protein-level data come from studies that either did not specify a subtype or examined combined cortical dysplasia and from those focused on FCD IIb. In contrast, mRNA investigations are largely concentrated on type II lesions (particularly IIa and IIb), while studies profiling miRNA dysregulation remain comparatively few and predominantly examine FCD IIb. This pattern highlights both the strong emphasis on type II lesions in molecular research and the relative scarcity of subtype-specific data for type I and type III FCD, underscoring an important gap for future studies.

### 3.2. Human-Specific Dysregulated miRNAs in FCD

**Figure 3:**
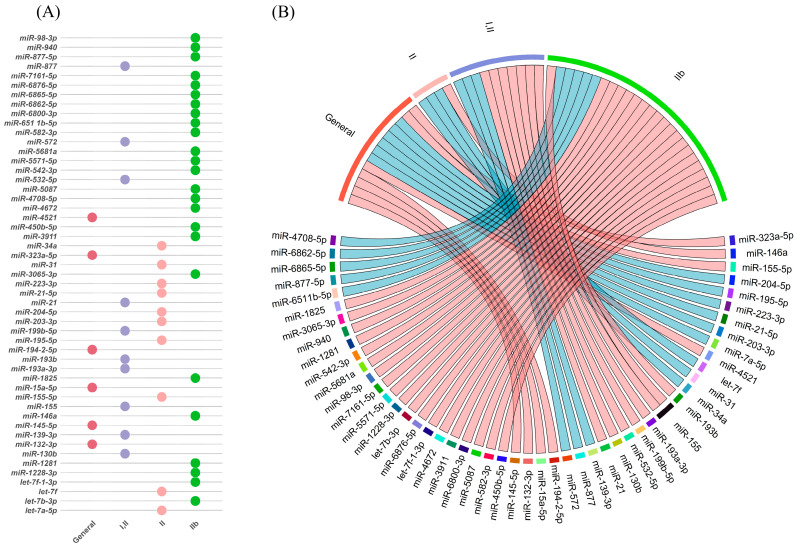
(**A**) Dot plot summarizing reported miRNA–FCD associations. Rows list unique miRNAs; columns show corresponding FCD types. Each colored dot marks a reported link between an miRNA and a specific subtype. (**B**) Circular chord diagram illustrating miRNA associations with FCD subtypes. Outer segments represent general (red) and subtype-specific groups: FCD I (purple), FCD II (green), and FCD III (blue). Connecting lines indicate dysregulation (light red = upregulated; light blue = downregulated).

This review identified eight articles investigating miRNAs in human FCD samples. These miRNAs are classified from two distinct perspectives: (1) a molecular and methodological approach, organizing them by expression status (upregulated or downregulated) and technique used (microarray and RT-PCR, RT-PCR only, or RNA-Seq and RT-PCR) (Table 1); and (2) a histopathological approach, classifying them by FCD subtype (Type I, II, or general/nonspecific FCD) (Figure 3). The supporting data are detailed in Supplementary File S3. Several miRNAs were reported as dysregulated in FCD samples, including hsa-miR-21-5p, hsa-miR-155-5p, hsa-miR-132-3p, and hsa-miR-223-3p. While many of these miRNAs showed consistency across studies, variability was observed based on FCD subtype and analytical platform.

### 3.3. Dysregulated RNA Expression in Human FCD

**Figure 4:**
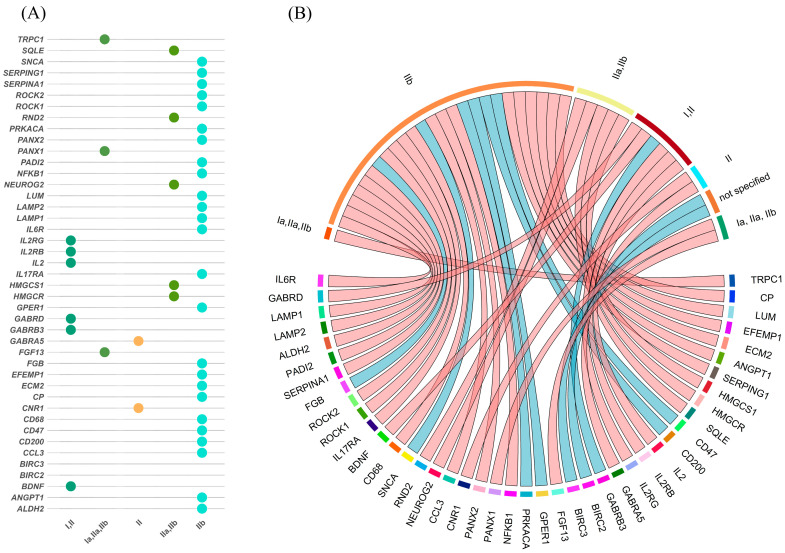
(**A**) Dot plot summarizing mRNA–FCD associations curated from the literature. Rows list genes reported in at least three studies; columns represent FCD types. Each colored dot marks a reported association, using the same FCD color scheme as in panel B. (**B**) Circular chord diagram showing mRNA associations with FCD subtypes (Ia, IIa, IIb). Lines connect genes (nodes) to their respective subtypes, with colors indicating regulation status (light red = upregulated; light blue = downregulated).

Analysis of 28 studies revealed significant alterations in mRNA expression associated with FCD. Results are categorized in two ways. First, by methodological approaches used for their identification in Table 2 that provides the reference, FCD subtype, and the specific RNAs identified as upregulated or downregulated. Second, by histopathological FCD subtypes (Type I, IIa, and IIb), as Figure 4 visualizes both shared and subtype-specific molecular changes. This dual categorization offers insights into the distinct pathophysiological mechanisms underlying each FCD subtype. The complete dataset supporting these classifications, including additional methodological details and references, is provided in Supplementary File S4.

Multiple genes were found to be dysregulated in FCD tissues, including *IL1B*, *TLR4*, *CD68*, *GABRA5*, and *ROCK1*. Differences between studies may reflect variability in patient populations, subtype definitions, and experimental platforms.

We analyzed overlapping mRNA expression patterns reported across independent studies to enhance our findings and reduce study-specific biases. Identifying these recurrent alterations improves the strength of our findings by ensuring consistent molecular patterns in FCD. We emphasize the most reproducible and biologically relevant molecular marker by focusing on RNAs reported as dysregulated in multiple investigations. Table 3 presents the RNA expression results consistently reported across studies, distinguishing upregulated and downregulated RNAs and categorizing them by FCD subtype when available. This overlap analysis offers a more integrated view of underlying FCD and helps prioritize candidate genes for future functional studies and potential therapeutic targeting.

### 3.4. Protein-Level Alterations in FCD Subtypes

**Figure 5:**
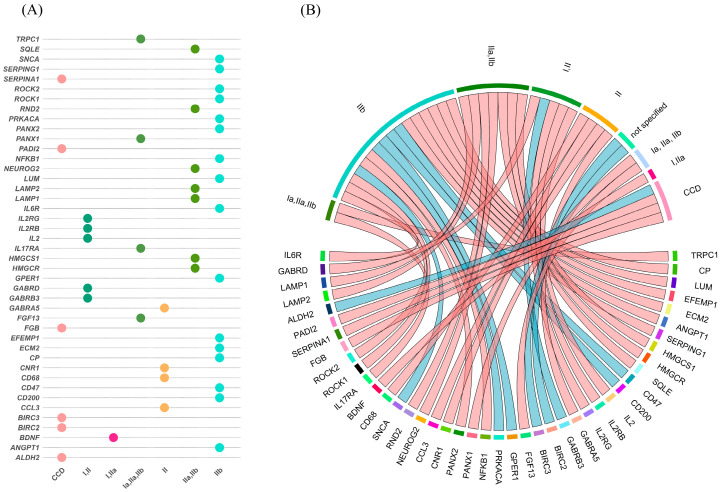
(**A**) Dot plot summarizing protein–FCD associations curated from the literature. Rows list proteins reported in at least three studies; columns represent FCD types. Each colored dot marks a reported association, using the same FCD color scheme as in the chord diagram. (**B**) Circular chord diagram showing protein associations with focal cortical dysplasia (FCD) subtypes and childhood cortical dysplasia (CCD). Outer segments represent FCD types and CCD categories. Lines connect proteins (nodes) to their respective categories, with link colors indicating regulation status (light red = upregulated; light blue = downregulated).

We reviewed 98 studies reporting protein in FCD. Results are organized according to the methodological approaches used to assess protein abundance. Studies were categorized into groups based on whether they used targeted techniques, such as immunohistochemistry (IHC), Western blotting (WB), and double-label immunofluorescence (DL-IF), or comprehensive, unbiased proteomic methods, such as mass spectrometry (iTRAQ). A detailed summary of dysregulated proteins, including their expression status (upregulated or downregulated), associated FCD subtypes, references, and methodologies, is provided in Table 4. Additionally, Figure 5 visually maps the distribution of protein alterations across FCD subtypes, showing both shared and subtype-specific molecular changes. Protein-level studies identified differential expression of markers such as IL1B, TLR4, GABRA5, and ROCK2 in FCD tissue. The majority of protein findings were based on immunohistochemistry or Western blotting, with a smaller number of mass spectrometry-based studies.

In addition, Table 5 summarizes proteins consistently reported across multiple studies to support the findings, indicating convergent molecular alterations in FCD. These overlapping findings strengthen the identification of shared molecular programs across FCD subtypes. The complete dataset, including all reported data and methodological details, is available in Supplementary File S5.

**Figure 6:**
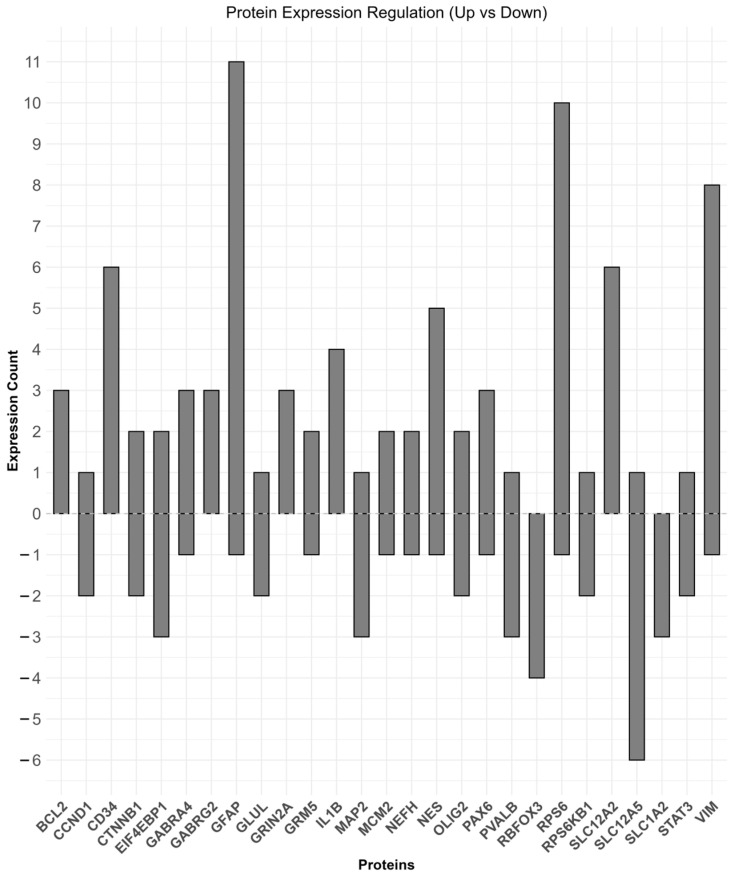
Summary of protein expression changes across studies in FCD. The bar plot shows the frequency of upregulation (positive values) and downregulation (negative values) for proteins identified across independent studies. Proteins involved in glial activation, synaptic function, and metabolic processes are demonstrated.

To further explain the consistency of protein abundance changes across studies, we summarized the direction of dysregulation (upregulation or downregulation) for proteins identified in multiple independent FCD investigations (Figure 6). Several proteins involved in key biological processes, such as GFAP and VIM, were consistently upregulated, suggesting an active role of gliosis in FCD pathology. In contrast, SLC1A2 and SLC1A3 were predominantly downregulated. This analysis emphasizes that despite methodological differences between studies, specific molecular alterations are recurrently observed across FCD subtypes. The consistent dysregulation of these proteins supports their potential as molecular markers and therapeutic targets in the FCD.

### 3.5. Overlap Between mRNA and Protein Expression Across FCD Subtypes

To address methodological variability and identify the most robust findings, we compiled consistently altered mRNA and protein datasets. These common dysregulations offer insight into epileptogenesis and may serve as therapeutic targets. Table 6 summarizes overlapping genes and corresponding proteins by FCD subtype, direction of expression change, and study references. This integrative analysis provides a coherent framework of the molecular network of FCD, supporting the design of future mechanistic studies.

### 3.6. Subtype-Specific Molecular Profiles

Analysis of differentially expressed genes across focal cortical dysplasia (FCD) showed different molecular signatures for Types I, II, and III. In FCD Type I, *GABRB3* was downregulated, whereas *GABRA1* was upregulated. FCD Type II exhibited the most extensive subtype-specific alterations, characterized by significant upregulation of *AKT1*, *FGF2*, *IL1B*, *TLR4*, *HMGB1*, *VIM*, *MYD88*, and *TRAF6*. In FCD Type III, the most prominently upregulated genes were *OLIG2* and *PAX6*.

## 4. Discussion

### 4.1. Molecular Pathways Enriched in FCD

**Figure 7:**
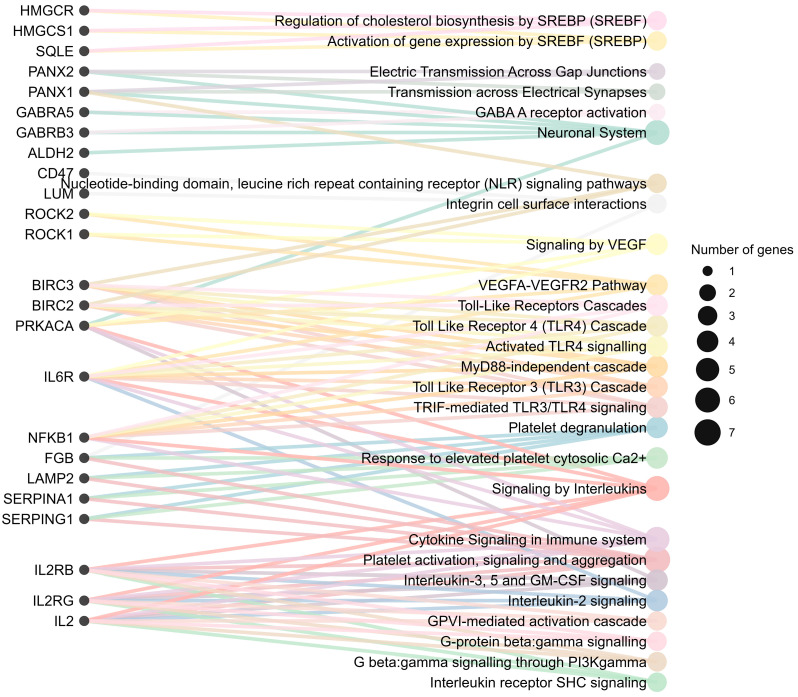
The Sankey diagram shows relationships between genes/proteins (left nodes) and associated biological pathways (right nodes). Line connections highlight shared and distinct functional associations.

Integrating transcriptomic and proteomic data across FCD subtypes reveals a coherent pattern across inflammatory signaling, synaptic transmission, cytoskeletal organization, and cellular metabolism (Figure 7). These signals are not isolated; they form an interconnected network that may sustain epileptogenesis. A cross-omics perspective helps distinguish subtype-restricted features from disease-wide pathways. As single-cell and spatial transcriptomic methods mature, bulk data can be mapped to specific cell types and cortical layers, clarifying contributions from dysmorphic neurons, reactive astrocytes, microglia, and the neurovascular axis. Connecting molecular profiles with clinical, histopathological, and outcome data should sharpen patient subtyping and guide mechanism-based therapy.

**Figure 8:**
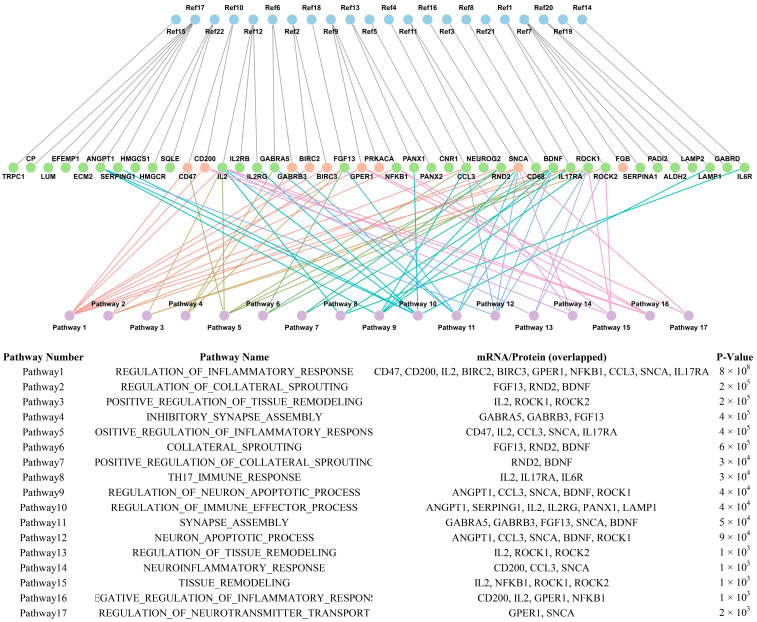
The network integrates transcriptomic and proteomic data to demonstrate targets consistently identified at both levels (middle layer), their associated enriched pathways (bottom layer), and supporting studies (top layer). Reference labels in the figure (Ref1–Ref22) correspond to the respective reference numbers in the text [106], [27], [44], [24], [29], [30], [117], [64], [33], [20], [31,35], [37,45], [93], [34], [38], [105], [114] and [47], respectively. This integrative analysis emphasizes convergent biological processes consistently supported across multiple studies.

We also looked at overlapping targets that were reliably found at the transcriptome and proteomic levels. The argument that these high-confidence targets represent basic biology rather than platform-specific artifacts is strengthened by the fact that they occupy the same convergent paths across several independent datasets, as illustrated in Figure 8.

#### 4.1.1. Inflammation and Immune-Mediated Mechanisms

Extensive evidence indicates that NF-κB is at the center of a persistent inflammatory program in FCD. As a canonical hub downstream of TLR/IL-1R/TNF receptors, NF-κB forces broad transcriptional outputs, *IL1B*, *IL6*, *TNF*, and chemokines (*CCL2/3/4*) that sustain leukocyte and microglial recruitment and facilitate tissue remodeling [122]. In our data, *TLR2/TLR4* and interleukin-signaling components are repeatedly upregulated alongside *IL1B/IL6/CCL2/3/4*, with protein-level markers of glial activation (*CD68* for microglia; *GFAP/VIM* for astroglia) consistently detected. Taken together, these findings indicate chronic NF-κB engagement within the dysplastic cortex, which is compatible with a self-reinforcing inflammatory state [122].

A second enhancer is *IL–17ra*-dependent signaling, which increases chemokine production and leukocyte trafficking in inflamed tissues and is expected to activate NF–κB–driven cascades in FCD [123]. More comprehensive neuroimmunology syntheses further emphasize *IL-1*, *IL-2*, *IL-6*, and *IL-17* as key coordinators of glial reactivity, barrier disturbance, and neuronal excitability, with vast cross-talk to PI3K–Akt–mTOR and metabolic control pathways [124].

This framework explains how the cytokine activity within lesions can drive growth, protein production, and metabolic changes that influence network stability. Notably, the immune checkpoint appears weakened in FCD IIb. Downregulation of CD47–SIRPα and *CD200–CD200R* removes inhibitory “brakes” on microglia, a configuration predicted to permit excessive synaptic pruning, cytokine release, and structural remodeling within lesions [33]. This checkpoint failure explains the stronger immune markers observed in IIb and aligns with the persistent glial activation captured across our transcriptomic and proteomic datasets. Overall, the data support neuroinflammation as a core, targetable axis of FCD, tightly grounded in the interleukin–NF-κB–TLR triad and reinforced by checkpoint loss in IIb [33,122,123,124].

#### 4.1.2. Neuronal and Synaptic Dysfunction

A key mechanistic feature of FCD is the failure of γ-aminobutyric acid (GABA)-mediated inhibition, which leaves cortical networks given to hyperexcitability. Sharma et al. [30] demonstrated that the dysplastic cortex maintains an immature chloride balance, characterized by reduced *KCC2* (*SLC12A5*) and increased *NKCC1* (*SLC12A2*) expression. This shift makes the neuronal chloride gradient less negative, so GABA_A_ receptor activation becomes depolarizing rather than hyperpolarizing, which lowers the seizure threshold and facilitates synchronous firing. At the same time, the subunit composition of GABA_A_ receptors are altered, with an increase in α5 (*GABRA5*) and δ (*GABRD*) subunits, which changes receptor kinetics and weakens tonic (extrasynaptic) inhibition. When chloride levels are abnormally high, these receptors can lose their inhibitory effect and even contribute to excitation. Our multi-omic synthesis supports and extends this mechanism: transcriptomic data across FCD II lesions consistently show downregulation of *SLC12A5* and upregulation of *SLC12A2*. At the same time, proteomic studies detect altered GABA_A_ receptor subunits, including increased *GABRA5* and *GABRD*. These patterns align closely with the lesion-level findings of Sharma et al. [30], indicating that defective chloride regulation and receptor remodeling are not isolated observations but reproducible molecular features across independent datasets. This convergence helps explain the persistent inhibitory failure seen across FCD subtypes and provides a molecular basis for the drug-resistant epileptogenic networks that define the disorder.

#### 4.1.3. Cytoskeletal Remodeling and Cell Migration

Convergent evidence suggests that cytoskeletal dysregulation is associated with disordered neuronal migration, polarity, and cortical lamination characteristic of FCD. Foundational studies on neuronal migration have shown that the precise coordination of microtubules and actin networks is essential for maintaining leading-process stability and establishing proper cortical layer formation; disruptions in these systems lead to persistent defects in lamination and connectivity [125]. Lesion-level data from FCD are consistent with this framework. *FGF13*, an intracellular fibroblast growth factor that supports microtubule stabilization and neuronal process organization, exhibits increased expression in the dysplastic cortex, consistent with a developmentally altered microtubule program that may persist into the mature lesion [40]. In parallel, *LILRB2* signaling is activated in FCD IIb, a pathway classically associated with growth-inhibitory signals and cytoskeletal remodeling at growth cones; such signaling is consistent with restricted neurite extension and atypical axon/dendrite architecture within dysplastic tissue [106]. These findings, together with the histopathological evidence of dysmorphic neurons and balloon cells in IIb, support the hypothesis that microtubule- and receptor-linked cytoskeletal programs fail to achieve standard migration/polarity, leading to an abnormal adjustment in mature lesions. As a result, abnormal circuit formation and miswiring lead to synchronized neuropathic activity and seizure spread, thus linking focal cortical dysplasia epilepsy to cytoskeletal disruption [40,106,125].

#### 4.1.4. Metabolic Dysregulation

Transcriptomic profiling of FCD type II consistently shows enrichment of cholesterol-biosynthesis pathways with increased expression of *HMGCS1*, *HMGCR*, and *SQLE*, key enzymes of the mevalonate pathway, indicating an upregulated lipid-synthetic state in dysplastic cortex [47]. This pathway regulates cholesterol and isoprenoid production, influencing membrane fluidity, lipid organization, and the trafficking of ion channels and synaptic vesicle proteins [126]. Such remodeling can disrupt neuronal signaling and lower the seizure threshold, helping maintain hyperexcitability.

Although best known in cancer [126], mevalonate-pathway activation in FCD likely reflects a broader metabolic reprogramming driven by chronic inflammation and growth signaling. In our dataset, these lipid changes co-occur with inflammatory and mTOR-related signatures, suggesting a coordinated control of membrane remodeling, synaptic vesicle cycling, and cell growth within lesions. This convergence highlights a potential therapeutic angle: normalizing cholesterol synthesis or membrane composition may help stabilize synaptic function and counteract epileptogenic network activity [47,126].

### 4.2. Crosstalk Between Inflammation, Metabolism, and mTOR in FCD

#### 4.2.1. Inflammatory Pathways and mTOR Activation

Pro-inflammatory cytokines converge on the PI3K–Akt–mTOR axis, establishing a mechanistic link between immune signaling and growth/remodeling in the dysplastic cortex. IL-6 suppresses the mTOR inhibitor *REDD1* through STAT3-dependent transcription, thereby facilitating negative regulation of mTOR [127]. Aberrant activation of the PI3K–Akt–mTOR pathway itself is a well-recognized molecular hallmark of FCD and related cortical malformations [128]. In parallel, *IL2* and other T-cell cytokines regulate mTOR to control cellular metabolism and proliferation [129]. In our data, these inflammatory markers co-occur with mTOR pathway output and with altered excitability, presented by *ASIC1a* downregulation within lesions [31], supporting the concept of cytokine-to-mTOR coupling in FCD. The PI3K–Akt–mTOR network functions as a hub for growth and survival signals [130] and can also respond to hemostatic signals present in epileptogenic tissue. Platelets promote seizures, enhance neuroinflammation, and increase oxidative stress in the brain, providing an additional input that can engage this axis [131]. Downstream, mTOR-driven transcription is required for neuronal cholesterol biosynthesis during cortical development [132], which relates to the inflammatory and platelet-derived factors that influence the lipid metabolic reprogramming observed in FCD lesions. Collectively, and consistent with frameworks placing mTOR at the core of FCD biology [133]. These findings support a model in which the PI3K–Akt–mTOR axis integrates cytokine and hemostatic inputs into metabolic and structural remodeling programs that sustain malformation dynamics and chronic hyperexcitability.

#### 4.2.2. Neuronal Signaling and Synaptic Dysfunction

Across FCD lesions, pathway analysis reveals increased involvement of GABA_A_ receptor–related signaling and altered receptor subunit composition, indicating a fundamental disturbance of inhibitory neurotransmission. Human studies have shown that GABA_A_ receptor mutations are associated with intracortical hyperexcitability, characterized by reduced inhibition and synchronized firing, providing direct evidence that receptor defects can destabilize cortical networks [134]. These inhibitory changes interact with PI3K–Akt–mTOR–dependent plasticity, which regulates protein synthesis, receptor trafficking, and synapse stability. In FCD, mTOR activation is well documented and offers a mechanism by which an initial inhibitory deficit can be stabilized and strengthened at the circuit level [135]. A second modulator is the BDNF/proBDNF system: mature BDNF enhances inhibitory tone, whereas proBDNF weakens it by altering synapse efficacy and chloride homeostasis; dysregulation can therefore shift networks toward excitation [136]. Together, these mechanisms support a self-reinforcing loop in FCD: GABA_A_ receptor dysfunction initiates hyperexcitability, while mTOR-driven plasticity and altered BDNF signaling strengthen and maintain epileptogenic circuits within an inflammatory, mTOR-active environment [137].

#### 4.2.3. Extracellular Matrix and Angiogenesis

Analyses in our dataset, which point to ECM remodeling in conjunction with VEGF/angiogenic pathway signals, indicate that vascular and extracellular components contribute directly to FCD pathophysiology. In particular, *ANGPT1* upregulation within lesion tissue is compatible with active angiopoietin–angiogenesis programs and structural vascular remodeling, changes that would be expected to alter extracellular space properties and local ion/neurotransmitter buffering, thereby shaping network excitability. Mechanistically, these vascular and matrix signals cross with mTOR-centered stress and growth pathways: in vascular systems, modulation of mTOR activity influences endothelial inflammatory tone, growth/repair programs, and angiogenic gene expression, providing a route by which lesion-level angiogenesis and ECM turnover can strengthen neuroinflammatory and metabolic shifts in FCD [138]. Taken together, the data support a neuro-glial-vascular model in which ECM remodeling and angiogenic signaling (including *ANGPT1*) help establish a tissue environment that sustains hyperexcitability: vascular changes reshape the extracellular environment and inflammatory signaling; these inputs converge on mTOR-linked metabolic and plasticity programs; and the resulting feedback stabilizes the epileptogenic state [138].

### 4.3. Integration of miRNA–Target Interactions in FCD

**Figure 9:**
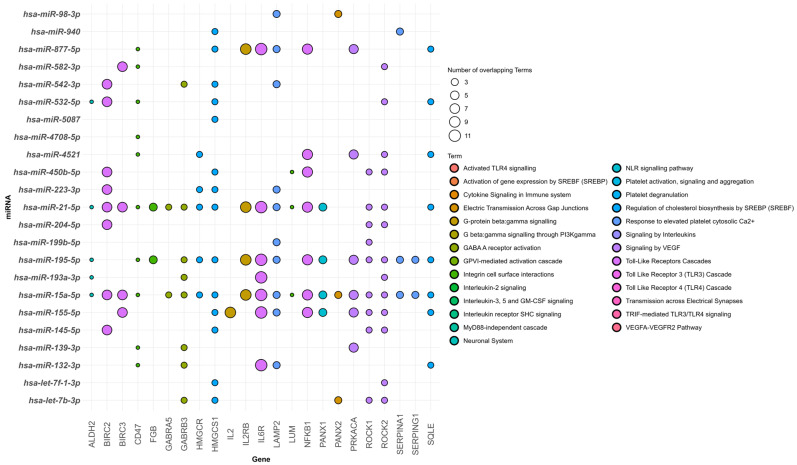
Bubble plot represents associations between microRNAs (*y*-axis) and their target genes (*x*-axis). Bubble size corresponds to the strength or frequency of interaction, and colors indicate functional categories or pathways.

The miRNA layer shows how different signals co-move in lesions by providing regulatory control across inflammatory, synaptic, metabolic, and vascular pathways. The main miRNAs and their verified targets are shown in Figure 9; we explain the most commonly reported species below:

miR-21-5p: Enriched in Toll-like receptor and interleukin signaling pathways and targets key inflammatory mediators, including *IL6R* and *BIRC3*. It is upregulated in both FCD Types I and II, where it contributes to immune modulation, microglial activation, and the maintenance of chronic neuroinflammation [21].

hsa-miR-155-5p: Regulates targets including *TLR4*, *NF-ΚB1*, *IL2*, and *BIRC3*, and is strongly linked to immune activation and apoptotic signaling. Its frequent upregulation in FCD Type II suggests a pivotal role in inflammation-driven dysplastic development [21,24].

hsa-miR-132-3p: By targeting *GABRB3* and *IL6R*, this miRNA links GABAergic dysfunction with neuroinflammatory pathways. It modulates synaptic plasticity and promotes cortical hyperexcitability by impairing inhibitory signaling [25].

hsa-miR-223-3p: Regulates metabolic targets such as *HMGCS1* and *HMGCR*, which are essential for lipid biosynthesis and cholesterol metabolism [139]. It also participates in Toll-like receptor and interleukin signaling by targeting key inflammatory genes. Its dysregulation in brain tissue shows its potential as a diagnostic biomarker in FCD [24].

hsa-miR-195-5p: This miRNA targets *ROCK1*, *BIRC2*, and *HMGCR*, setting it at the intersection of apoptosis, metabolic regulation, and platelet activation. By additionally regulating *GABRB3*, it contributes to synaptic transmission and vascular remodeling, suggesting a multifaceted role in FCD pathology [24].

hsa-miR-15a-5p: It targets pro-apoptotic and inflammatory genes, including *BIRC2* and *ROCK1*, implicating it in neuronal survival and cytoskeletal regulation [25].

Together, these miRNAs provide circulating biomarkers and regulatory nodes for intervention by coordinating immunological, neural, vascular, and metabolic programs.

### 4.4. Clinical Implications

The recurrent molecular alterations identified in this review, particularly inflammatory mediators, enzymes involved in cholesterol biosynthesis, and consistently dysregulated miRNAs, represent promising candidates for clinical translation. Several of these miRNAs have been detected not only in resected cortex but also in serum and exosomes, with early studies suggesting their potential as non-invasive biomarkers for focal cortical dysplasia. A practical path to clinical use involves technical confirmation in independent surgical and biofluid samples using standardized, reproducible assays with strict pre-analytical control; clinical validation in large, multicenter cohorts with careful documentation of age at seizure onset, epilepsy duration, and brain region, together with external validation to ensure findings generalize across centers; and clinical utility testing, where validated molecular panels are combined with neuroimaging and electrophysiology to improve detection of lesions that are histopathologically proven but not visible on standard MRI, guide surgical or minimally invasive ablation planning, predict post-operative seizure freedom, and monitor treatment response to targeted interventions such as anti-inflammatory or metabolic therapies. Embedding these steps within prospective, harmonized protocols and sharing data through multicenter networks could accelerate the translation of molecular insights into reliable diagnostics and mechanism-based treatment strategies for drug-resistant FCD.

### 4.5. FCD Type-Specific Insights

FCD Type I: *GABRB3* downregulation and *GABRA1* upregulation indicate impaired inhibition consistent with hyperexcitability [30], aligning with enrichment of GABAergic dysfunction in this subtype.

FCD Type II: Altered *AKT1*, *FGF2* [140], and inflammatory mediators (*IL*1B [141], *TLR4*, *HMGB1*, *MYD88*, and *TRAF6* [42]) indicate coordinated activation of growth and immune–gliotic pathways. Upregulation of *VIM* integrates with PI3K–Akt–mTOR and TLR/cytokine signals, consistent with IIb histopathology [46].

FCD Type III: Upregulation of *OLIG2* and *PAX6* suggests disruption of neurogenesis and progenitor cell dynamics [71]; *OLIG2* is essential for oligodendrocyte development/myelination [142] and PAX6 for cortical patterning [143]. Subtype-specific abnormalities may be caused by these gene-level changes that correspond to neurodevelopmental, glial-precursor, and myelination pathways.

Together, these patterns show that while neuroinflammatory, synaptic, cytoskeletal, and metabolic pathways converge across FCD types, others are subtype-dependent, reflecting different pathogenic drivers.

### 4.6. Age-Related Molecular Heterogeneity in FCD

A convergent set of studies indicates that the molecular landscape of FCD varies with the developmental stage at seizure onset. At the transcript and receptor levels, early-onset lesions keep an immature inhibitory phenotype characterized by a disrupted *NKCC1*-*KCC2* balance and GABA_A_ subunit composition (α5/δ), which favors depolarizing GABA. In contrast, late-onset cases exhibit milder changes [30]. Pediatric FCD IIb exhibits *IL1β* potentiated, depolarizing GABA currents with *NKCC1* upregulation, while adult IIb shows the opposite *IL1β* modulation [48]. Protein-level data suggest age-linked cellular state transitions: in IIb balloon cells, children display G1/S licensing markers (*Cdk2*, and *Cdk4*), whereas adults show loss of Rb consistent with greater cell-cycle arrest [54]. In drug-resistant temporal lobe epilepsy associated with FCD, adults exhibit higher GFAP/S100/caspase-3, while children show more vimentin, with region-dependent patterns [68]. Matrix metalloproteinases are more prominent in adult FCD and rarely detected in children [103]. Developmental-lineage markers also show gradients: DCX-positive immature neurons are abundant in very young FCD Ia and decline with age, whereas aberrant *DCX* persists in IIb dysmorphic/balloon cells into adulthood [109]. Collectively, these data support a trajectory in which younger/early-onset lesions are enriched for neuronal immaturity and excitatory-shifted inhibition. In contrast, older/long-standing lesions exhibit matrix remodeling, inflammatory, and cell cycle arrest features. Nevertheless, most studies have neither stratified nor adjusted for age and often use age-mismatched controls, limiting quantitative age-adjusted synthesis. We, therefore, present the whole output to preserve completeness, emphasize the age-analyzed exemplars, and recommend standardized reporting of age at seizure onset, age at surgery, epilepsy duration, age-matched controls, and age-adjusted/stratified analyses in future FCD molecular work.

### 4.7. Region- and Compartment-Related Molecular Heterogeneity in FCD

Most included studies specified the lobe sampled (predominantly temporal or frontal), but only a few incorporated regional or gray–white compartmentalization into molecular analyses. Protein-level studies demonstrate that anatomical context is significant. In drug-resistant temporal lobe FCD, adults exhibit higher levels of *GFAP*, *S100*, and caspase-3, while children show increased vimentin levels, with more potent effects in the cortex than in the white matter [68]. Guo et al. [84] reported aberrant adenosine signaling with distinct gray–white distributions, where *ADA/ADK/NT5E/ADORA2A* were predominantly increased in gray matter dysmorphic neurons. In contrast, reactive astrocytes in the white matter also contributed. Li et al. [85] found lobe-dependent changes in neuropeptide-Y receptor expression (Y1R/Y2R were enriched in temporal/frontal regions, but not in the parietal areas, with Y5R/NPY increased more broadly). Gruber et al. [101] demonstrated that complement activation was stronger in white matter with synaptic loss in gray matter in FCD IIb. Together, these data suggest that microenvironmental context (lobe, cortical layer, gray–white interface) shapes immune and neurotransmitter-related signaling in FCD. Nevertheless, most studies either pooled all lobes or did not stratify by compartment, preventing quantitative, region-adjusted synthesis. Standardized reporting of sampled lobe, gray/white composition, and lesional vs. perilesional zones will be essential for future cross-study comparisons.

### 4.8. Integration with Recent Single-Cell and Spatial Transcriptomic Studies

Our systematic review synthesizes bulk multi-omic data (miRNA, mRNA, protein) across focal cortical dysplasia (FCD) subtypes. Recent single-cell and spatial transcriptomic studies now provide crucial cell-type–resolved context for these convergent pathways. In FCD II, multimodal single-nucleus profiling shows a selective loss of upper-layer excitatory neurons and the emergence of disease-specific dysmorphic excitatory neurons, accompanied by immature astrocytes and activated microglia, particularly in type IIb [144]. Genotype single-cell analyses demonstrate that somatic PI3K–mTOR pathway variants occur in both differentiated neurons and glia. Variant excitatory neurons upregulate mTOR and metabolic programs, while neighboring non-variant neurons downregulate these pathways and alter glutamate/GABA_A_ signaling [145,146].

Spatial transcriptomics in type IIb lesions further localizes mTOR/ubiquitin proteasome and membrane potential related to dysmorphic neurons, while complement and inflammatory programs are enriched in balloon cell areas, validated at the protein level [147]. In FCD IIIa, single-nucleus multi-omics has identified a DAB1-high excitatory neuron subpopulation with immune-related transcriptional profiles and activated glial precursor cells with disrupted neuron–glia signaling, aligning with our bulk findings of immune activation and myelination changes [148]. Additional single-nucleus profiling in IIb has described neurovascular remodeling, including ischemic–hypoxic microenvironments that trigger HIF-1α/mTOR/S6 activation, astrocyte dysfunction, and neuronal loss, supported by experimental seizure models [149]. Very recent spatial mapping further extends these insights: Wang et al. (2024) [147] localized mTOR/ubiquitin–proteasome and inflammatory programs specifically to dysmorphic neurons and balloon-cell niches in IIb, integrating transcriptomic patterns with protein validation and lesion microarchitecture.

Together, these high-resolution datasets confirm and refine the neuroinflammatory, synaptic, cytoskeletal, and metabolic alterations we identified at the bulk level, map them to defined cortical layers and cell populations (dysmorphic neurons, balloon cells, reactive astrocytes, microglia, and vascular cells), and link somatic mTOR activation and neurovascular stress to epileptogenesis. Single-cell and spatial approaches, therefore, complement bulk multi-omics, helping to localize shared molecular networks, explain subtype-specific heterogeneity, and highlight actionable cellular targets for precision therapies.

### 4.9. Limitations

#### 4.9.1. Biological and Methodological Heterogeneity

Heterogeneity in FCD studies derives from both biological and methodological factors. Earlier work often used the Palmini classification, while more recent studies applied the ILAE criteria, complicating subtype comparisons and potentially misaligning molecular profiles. Patient-related variables such as age at surgery and lesion localization also influence transcriptomic and proteomic findings through differences in cortical maturation, connectivity, and cell composition. Tissue quality varies as well, affecting RNA and protein integrity. Bulk tissue analysis is further confounded by pathological changes and uneven evidence across subtypes, with type IIb disproportionately represented.

Methodological diversity adds another layer of variation. Transcriptomic studies used RNA-seq, microarrays, or RT-qPCR, while proteomic studies applied LC–MS/MS/MS, 2D-DIGE, IHC, or Western blotting, each differing in sensitivity, coverage, and quantitative accuracy. Even within the same platform, divergent statistical thresholds and bioinformatic pipelines contribute to inconsistent findings. As a result, specific pathways may appear significant in some studies but absent in others due to detection limits, sampling bias, or cohort composition.

These factors limit strict meta-analysis; therefore, we emphasize qualitative, stratified convergence rather than pooled effect sizes. Despite this heterogeneity, consistent dysregulation of neuroinflammatory and synaptic pathways across datasets suggests strong shared mechanisms that require a better study design.

#### 4.9.2. Variability in Sample Type and Preservation

Across studies, sample types varied widely, including brain tissue, serum exosomes, and induced pluripotent stem cells (iPSCs). Preservation methods also differed (fresh, frozen, or formalin-fixed), each with distinct impacts on RNA and protein stability. Such differences in sample origin and handling influence expression levels and detection reliability, further complicating cross-study comparisons. Age was commonly reported but rarely integrated into analyses; only a small subset of studies examined age effects, and controls were frequently age-mismatched, limiting quantitative age-adjusted synthesis. In addition, the brain region was commonly stated (most often the temporal or frontal cortex) but inconsistently defined, and only a few studies analyzed the effects of the region or gray–white compartment. This heterogeneity, together with the frequent pooling of lobes and the lack of region-matched controls, prevented a formal lobe-specific or compartment-adjusted meta-analysis.

#### 4.9.3. Publication Bias and Incomplete Reporting

There is a potential for publication bias toward positive results. In addition, many studies lacked complete reporting of key statistical details such as *p*-values, effect sizes, variance/dispersion, and multiple-testing adjustments. They often presented incomplete quantitative data—particularly in protein studies using IHC or Western blotting. Gene/protein identifiers were not always standardized across studies, and raw data were not universally available, complicating harmonization. These limitations interfere with the ability to perform strict meta-analyses and weaken the overall strength of combined findings.

#### 4.9.4. Reliance on Histopathological Diagnosis

Most molecular studies of FCD are based on histopathological diagnosis, which can vary between observers. Overlaps between specific FCD subtypes and other brain conditions, such as tumors or inflammatory lesions, can make it harder to detect subtle but critical molecular differences. This uncertainty, combined with the historical shift from the Palmini to the ILAE classification system, continues to slow progress toward more accurate molecular classification and more targeted treatments for each subtype.

Despite these limitations, a key strength of this review is its ability to present diverse and inconsistently reported multi-omic findings side-by-side, using standardized identifiers and adjusted summaries. This integrated approach not only emphasizes convergent neuroinflammatory, synaptic, cytoskeletal, and metabolic pathways but also makes existing evidence gaps clear. In addition, it provides a framework for future research, encouraging larger cohorts, more explicit subtype definitions, and more consistent reporting that can directly build on this synthesis.

## 5. Conclusions

This systematic review integrates microRNA, RNA transcript, and protein data to demonstrate convergent molecular pathways in FCD, including Toll-like receptor signaling, GABAergic transmission, *VEGFA–VEGFR2* signaling, and lipid biosynthesis. Dysregulated microRNAs such as hsa-miR-223-3p, hsa-miR-132-3p, and hsa-miR-155-5p regulate key targets including *IL6R*, *ROCK2*, *PANK2*, *GABRB3*, and *SERPING1*, emphasizing roles in immune regulation, neuronal plasticity, and metabolism. These findings suggest that combining molecular markers with neuropathological assessment may improve precision medicine strategies in FCD. Importantly, this review provides the first cross-omics molecular framework of FCD, reframing heterogeneous findings into a unified model of pathogenesis and establishing a roadmap for biomarker discovery and therapeutic development.

### State of the Field and Future Research and Recommendations

The recent International League Against Epilepsy (ILAE) consensus has stabilized the classification of FCD and encouraged a multi-layered approach to diagnosis, integrating pathology, imaging, and genetics. Genetically, lesion-confined mTOR-pathway mosaicism accounts for a substantial subset of FCD type II, while *SLC35A2* mutations underlie mild malformation of cortical development with oligodendroglial hyperplasia in epilepsy (MOGHE). Other FCD types remain genetically heterogeneous and are frequently MRI-negative or radiologically subtle.

Detection of such subtle lesions is improving through machine learning-based analysis of structural MRI, often using surface-based features to emphasize dysplasia. These approaches perform best when models are interpretable and trained on large, shared, multicenter datasets; however, routine clinical adoption will require strong external validation and calibration.

Therapeutically, surgical resection remains the mainstay. Minimally invasive options, such as laser interstitial thermal therapy (LITT) and stereo-EEG-guided radiofrequency thermocoagulation (SEEG-RFTC), extend treatment to the profound or eloquent cortex with favorable safety profiles and seizure outcomes, provided that candidate selection, anatomical targeting, and completeness of ablation are carefully managed.

A central biological challenge is detecting ultra-low-variant–allele–fraction somatic mosaicism, which can evade standard assays. Targeted capture, cell-type-focused or ultra-deep sequencing, and DNA recovered from explanted SEEG electrodes may improve detection, particularly in MRI-negative or deep lesions.

Key methodological bottlenecks remain: age and brain region are rarely modeled despite clear biological relevance; protocols and metadata are heterogeneous; and many analytic pipelines lack external validation. Future progress will likely come from age- and region-aware study designs with harmonized sampling and transparent statistics; integrative multi-omics coupled to single-cell and spatial approaches and quantitative proteomics to localize signals to dysmorphic neurons, balloon cells, and reactive glia; multicenter biobanks enabling pooled re-analysis; and interpretable, externally validated machine-learning tools embedded in presurgical workflows. Mechanism-linked human models (acute surgical slices and patient-derived systems) could test whether modulating inflammatory and mTOR programs restores chloride homeostasis (*NKCC1–KCC2* balance) and normalizes network physiology. Future cohorts integrating circulating microRNAs, including exosomal microRNAs, with advanced MRI may support the development of multi-analyte, non-invasive biomarker panels for diagnosis, monitoring, and stratified, mechanism-guided care across all FCD types.

## Figures and Tables

**Table 1:** Summary of studies analyzing miRNA expression in FCD. This table lists references, FCD subtypes, sample types, and upregulated or downregulated miRNAs. A dash (“-”) indicates that no data were reported. The studies are categorized based on methodological differences.

FCD Type	Upregulated	Downregulated	Age (Year)	Sample Type	Reference
microarray and RT-PCR techniques					
General	hsa-mir-323a-5p	-	37–61	Temporal cortex	[18]
General	hsa-miR-4521	-	37–61	Temporal cortex	[19]
IIa, IIb	-	hsa-let-7f hsa-mir-31 hsa-mir-34a	2–18	Temporal and frontal cortex	[20]
I, II	hsa-mir-21 hsa-mir-155 hsa-mir-193a-3p hsa-mir-130b	hsa-mir-139-3p hsa-mir-877 hsa-mir-572	3–19	Frontal cortex	[21]
IIb	hsa-miR-1825 hsa-miR-3065-3p hsa-miR-940 hsa-miR-1281 hsa-miR-5681a hsa-miR-98-3p hsa-let-7b-3p hsa-let-7f-1-3p	hsa-mir-6511b-5p hsa-mir-877-5p hsa-mir-6865-5p hsa-mir-6862-5p hsa-mir-4708-5p	4–9	Frontal cortex	[22]
RT-PCR technique					
IIb	hsa-mir-146a	-	1–16	Frontal and temporal cortex	[23]
II	hsa-mir-155-p	hsa-mir-223-3p hsa-mir-21-5p hsa-mir-204-5p hsa-mir-195-5p hsa-mir-203-3p hsa-let-7a-5p	4–51	cortex	[24]
RNA sequencing (RNA-Seq) with RT-PCR validation techniques					
General	hsa-mir-194-2-5p hsa-mir-15a-5p hsa-mir-132-3p has-mir-145-5p	-	2–22	Serum exosomes	[25]

**Table 2:** Summary of studies using different techniques to assess RNA expression changes in FCD. This table lists references, FCD subtypes, and the upregulated or downregulated RNAs identified. A dash (“-”) indicates no data reported for that category. Age is expressed in months (m) or years (y).

FCD Type	Upregulated	Downregulated	Age	Sample Type	Reference
RT-PCR technique					
IIb	*TLR2*, *TLR4*, *AGER*	-	11–26 y	Temporal, Frontal	[26]
IIb	-	*BIRC2*, *PIK3CA*, *CTNNB1*, *EIF4EBP1*, *BIRC3*	12–45 y	Frontal and left orbitofrontal	[27]
IIb	*HMOX1*, *FTH1*, *ELANE*, *ALDH2*, *ELANE*, *FTH1*	-	4–18 y	Frontal and temporal lobe	[28]
II	*IL1B*, *IL6*, *CCL3*, *CCL4*, *STAT3*, *JUN*, *CCR5*	IL10	4–51 y	Frontal, temporal	[24]
II	*CNR1*, *RPS6*	-	3–21 y	Frontal and temporal cortex	[29]
I, II	*GABRA1*, *GABRA5*, *GABRG2*, *NKCC1*, *GABRA4*, *GABRD*	*GABRB3*	0–9 y and ≥10 years	Frontal and temporal cortex	[30]
I, II	-	*ASIC1*	4.8–7.1 y	Frontal, temporal	[31]
IIb	*NF-ΚB1*, *IL6*, *IL1B*	*GPER1*, *PRKACA*	4–35 y	Cortex not consistently specified	[32]
IIb	*-*	*CD47*, *CD200*, *SIRPA*, *IL4*	1.8–9.5 y	Frontal and temporal	[33]
IIa, IIb	*NEUROG2*, *RND2*	*-*	2–18 y	Temporal and frontal	[20]
Ia, IIa, IIb	*TRPC1*	*-*	1.2–12 y	Frontal, temporal, parietal, occipital	[34]
I, II	*IL2*, *IL2RA*, *IL2RB*, *IL2RG*	*-*	1–11.5 y	Frontal, Temporal, Parietal, Occipital	[35]
I, II	*TRPC6*, *BDNF*	*-*	2–33 y	Frontal, Temporal	[36]
Ia, IIa, IIb, IIb	*I: PANX1* *II: PANX1*, *PANX2*	*-*	1–7 y	Frontal, Temporal	[37]
IIb	*GRIN2A*, *GRIN2B*	*SNCA*	3.5–34 y	Frontal, temporal, parietal	[38]
Ia, IIa, IIb	*P2RX7*, *IL1B*	*-*	1–20 y	Frontal, Temporal, Parietal, Occipital	[39]
Ia, IIa, IIb	*FGF13*	*-*	1–19 y	Frontal, Temporal, Parietal, Occipital	[40]
I, II	*I*, *II: GABRG2*, *GABRD* *II: GABRA4*,	*-*	3–32 y	Frontal, Temporal, Parietal, Occipital	[41]
IIa, IIb	*TLR4*, *IL1B*, *TNF*	*-*	1–13 y	Frontal, Temporal, Parietal	[42]
IIb	*β-actin*, *BF-1/FOXG1*, *ErbB3*, *CaMKII*, *CREB*, *IGF-1*, *IGF-2*, *OTX-1 and NOS*	*BMP-6*, *EGFR*, *TGF-βR1*, *TGF-βR3*, *TGF-β1/2*, *c-fos and HES1*	1–20 y	Frontotemporal	[43]
RNA-Seq techniques					
IIa, IIb	*HLA-DRA***,***HLA-A***,***CD68*, *CCL2*, *CCL19*, *NOS2*, *C1q*, *C3d*	80 genes downregulated but not validated	2–29 y	Frontal and temporal	[44]
IIa, IIb	IIa, IIb*: CP*, *LUM*, *TNC*, *MANT2*, *EFEMP1*, *ECM2*, *ANGPT1*, *SERPING1* IIb*: CHI3L1*, *CCL2*	-	not provide	Neocortex from temporal	[45]
IIb	*GFAP*, *VIM*, *NES*, *S100B*, *C3*, *SERPINA3*, *STAT3*	-	1–40 y	Frontal and temporal	[46]
IIa, IIb	*IIa and IIb: HMGCS1*, *HMGCR*, *SQLE.* *IIa: MTRNR2L12* *IIb: GPNMB*	-	1–50 y	Frontal	[47]
IIb	*IL1B*, *IL1RN*, *SLC12A2/NKCC1* in Pediatric not adult	-	Adult > 18 y and Pediatric < 12 y	Temporal, parietal, and frontal	[48]
Microarray and ISH techniques					
IIa, IIb	*NEFH*	-	3–16 m	Frontal, Temporal, Parietal	[49]
IIa	-	*KCC2* (in small dysplastic neuron)	8–37 y	Frontal	[50]

**Table 3:** The consistently dysregulated RNAs, categorized by FCD subtypes, where available. In this table, “+” indicates increased expression.

Publication							
[26]		+	+				
[42]	+		+				
[48]	+						
[28]				+			
[24]	+						
[39]	+						
[32]	+						
[46]					+		
[43]						+	
[41]							+
[30]							+
	*IL1B*	*TLR2*	*TLR4*	*ELANE*	*C3*	*ERBB3*	*GABRA4*
Type			IIb		II		Mix
Color							

**Table 4:** Overview of dysregulated proteins in FCD. This table lists references, FCD subtypes, and upregulated or downregulated proteins. A dash (“-”) indicates no data reported. Age is expressed in months (m) or years (y).

FCD Type	Up Regulated	Down Regulated	Age	Sample Type	Reference
Immunohistochemistry (IHC) Techniques					
I, II	ASCL1, PROX1, TUBB3	MAP2	6–19 m	Parietal, frontal, temporal, occipital	[51]
IIa, IIb	NKCC1	GAD67, KCC2	3–54 y	Parietal, frontal, temporal, occipital	[52]
IIb	-	KCC2 in neuropil/mislocalized to soma	3–31 m	Frontal, temporal, parietal	[53]
IIb	MCM2	GMNN, CCNE1, CCND1, CDK2, CDK4, RB1	1–81 y	Frontal, parietal, temporal, and multilobar lesions	[54]
IIb	-	BIRC2, IK3CA/B, CTNNB1, EIF4EBP1, BIRC3	12–45 y	Frontal and left orbitofrontal	[27]
IIa, IIb	HLA-I/II, CD3, CD8, CD68, TSPO, CCL2, CCL19, C1q, C3d, COX-2, and IL-17 upregulated in FCD (stronger in IIb)	-	2–29 y	Frontal and temporal lobes	[44]
Ia, IIIa	PVALB, CALB1, CALB2	-	~25 y	Temporal	[55]
IIb	CASP3, CASP6, APP, TNFRSF21, SQSTM1, pS6 MAPT	-	23–50 y	Temporal (most frequent), frontal, and some parietal	[56]
II	SOX2, KLF4, RPS6	-	2–14 y	Mostly frontal	[57]
IIa, IIb	IIa, IIb: GFAP, CRYAB, IIb: CD34	-	IIa: 3 my–7 y IIb: 4.2–16	Frontal, occipital	[58]
Ia, Ib, IIa, IIb	-	PVALB	1–51 y	Temporal parietal, frontal	[59]
IIb	FABP7, VIM, ASCL1, SLC17A7, SLC17A6, PAX6, GFAP	SLC32A1, DLX1, DLX2	1–15 y	mostly temporal but also parietal, frontal,	[60]
I, III	-	PVALB	~38 y	Temporal, frontal, parietal and occipital	[61]
IIb	VIP	PVALB	0.4–26 y	Frontal, temporal, parietal, and occipital	[62]
IIa, IIb	PSMB9, PSMB8, PSMB6, PSMB5	-	18–45 y	Frontal, temporal	[63]
IIa, IIb	TRPC4, PLCD1	-	1.5–12 y	Frontal, temporal, parietal, and occipital	[64]
II	CD3E, CD8A, HLA-DRA, C1QA, C3, IL1B, CCL2	-	1–17 y	Temporal neocortex	[65]
IIb	GRM5, GRM1, GRM2, 3 (glia expressions)	GRM2, 3 in neuronal expressions	1–50 y	Majority were frontal, several parietal, and temporal	[66]
IIa, IIb	ABCB1, ABCC1	-	1–51 y	Frontal, temporal, parietal, and occipital	[67]
I, II, III	GFAP, VIM, S100, CASP3	-	1–45 y	Temporal lobe cortex and white matter	[68]
IIb	VIM, BCL2, PROM1, NES, MAP2, GFAP, TUBB3	RBFOX3	1–12 y	Frontal neocortex	[69]
II	BCL2L1, BAX, BCL2, TP53	-	6 weeks–57 y		[70]
I, II, III	GFAP, MCM2, PAX6, OLIG2, PDGFRB with subtype-specific differences (GFAP/PAX6 in FCDIIIa, Olig2 and GFAP/MCM2 in FCDIIIb, PDGFRβ in FCD1a	-	3–47 y	Temporal, frontal, occipital	[71]
IIb	CHI3L1, CCL2	-	not provide	neocortex from temporal lobe	[45]
IIb	GFAP	MAP2	1–40 y	Frontal and temporal lobes	[46]
IIb	PROM1, NES, VIM, GFAP	-	8–55 y	Temporal, Frontal	[72]
IIb	GFAP+1	-	27.3 ± 12.7 y	Temporal, Frontal	[73]
IIb	FGF2	-	2–45 y	Temporal, Frontal	[74]
IIb	FGF2	-	11 weeks–45 y	Temporal, Frontal	[75]
I, II	I, II: GABRG2 GABRA4 (markedly higher in II)	-	3–32 y	Frontal, Temporal, Parietal, Occipital.	[41]
I, II	I, II: RPS6, II: EIF4EBP1	-	10 weeks–49 y	Cortex	[76]
IIa	-	KCC2 (in small dysplastic neuron)	8–37 y	Frontal	[50]
IIa, IIb	-	GRM5	19–56 y	Frontal, parietal, temporal, para hippocampal, orbitofrontal, fusiform gyrus	[77]
IIb	CCND1, RPS6, CTNNB1	STAT3, EIF4EBP1, RPS6KB1	1–20 y	Temporal, Frontal	[78]
IIa, IIb	IIa, IIb: GFAP, HLA, IIb: CD3, VIM	RBFOX3	1–34 y	Temporal, Frontal	[79]
I, II	II, I: TBR1, OTX1, PAX6, MAP1B, II: ER81, NEFM	-	1–52 y	Temporal, Frontal	[80]
Ia, Ib, IIa, IIb	-	SLC1A3, SLC1A2	2–44 y	Lateral temporal neocortex	[81]
II	INA, NES, NEFL, NVIM, NEFM, NEFH, PRPH	-	15–30 y	temporal neocortex	[82]
IIa, IIb	IIa, IIb: HMGCS1, IIb: HMGCR, SQLE, GPNMB	-	1–50 y	Frontal	[47]
IIb	RPS6		1–20 y	Frontal, temporal	[43]
IIa, IIb	IIa, IIb: RPS6, WNT, RPS6KB1, IIb: NES, EIF4EBP1, SOX2, CD34	CCND1, CTNNB1 Not detected	1–33 y	Frontal and temporal	[83]
Immunohistochemistry (IHC) and Western Blotting (WB) Techniques					
I, II	ADA, ADK, NT5E, ADORA2A	SLC1A2	6–27 y	lesional cortex	[84]
I, II, III	NPY2R, NPY1R, NPY5R, NPY	-	2–41 y	Temporal, Frontal, Parietal	[85]
IIa, IIb	IIa, IIb: SLC12A2, IIa: SLC12A5	IIb: SLC12A5 IIa: GABRA4, IIb: GABRA1	~10.3 y	neocortex	[86]
IIb	VEGFB, VEGFA, FLT1, KDR	-	11–31 y	Temporal lobe	[87]
IIb	HMOX1, NFE2L2, FTH1, FTL	-	4–18 y	Frontal, temporal	[28]
I, IIa	BDNF, NGF, NTF3	-	6–36 y	temporal	[88]
IIb	DCLK1	-	11–41 y	Frontal, temporal	[89]
IIa, IIb	CTNNB1	NR1H2, FABP7, PDGFRA, OLIG2	1.5–18 y	Frontal, temporal, parietal, occipital	[90]
II, I	-	ASIC1	4.8–7.1 y	Frontal, temporal	[31]
IIb	NF-ΚB1	GPER1, PRKACA	4–35 y	cortex	[32]
IIb	-	CD47, CD200, SIRPA	1.8–9.5 y	Frontal and temporal	[33]
IIa, IIb	TRPC4, PLCD1	-	1.5–12 y	Frontal, temporal, parietal, and occipital	[64]
Ia, IIa, IIb	TRPC1	-	1.2–12 y	Frontal, temporal, parietal, and occipital	[34]
I, II	IL2, IL2RA, IL2RB, IL2RG	-	1–11.5 y	Frontal, temporal, parietal, and occipital	[35]
IIb	KCND2, P- KCND2, pERK1/2	-	14–41 y	Frontal, temporal, parietal	[91]
Ia, IIa, IIb	TRPC3	-	<12 y	frontal, temporal, parietal, occipital	[92]
Ia, IIa, IIb	Ia, IIa, IIb: PANX1, IIb: PANX2	-	1–7 y	Frontal and temporal	[37]
IIb	p-STAT3, IL6, IL6R, JAK2	-	1.2–9 y	frontal, temporal, parietal	[93]
IIb	RTN4R, LINGO1, TNFRSF19, RTN4, RHOA	-	1.2–8.5 y	frontal, temporal, parietal, occipital	[94]
IIb	-	SV2A	12–40 y	frontal, temporal	[95]
IIb	GRIN2A, GRIN2B	SNCA	3.5–34 y	Mainly frontal	[38]
IIa, IIb	FLNA, RPS6	-	1–14 y	frontal, temporal	[96]
IIa, IIb	GAP43	-	2.6–14 y	frontal, parietal, temporo-parieto-occipital	[97]
Ia, IIa, IIb, IIIa	-	KCC2	7–48.5 y	frontal, temporal	[98]
IIb	RPS6, VIM	MBP, CNP, PDGFRA	1–18 y	frontal, parietal, temporo-parieto-occipital	[99]
Ia, IIa, IIb	TRPV4, PRKCA	-	1.5–12 y	frontal, parietal, temporo-parieto-occipital	[100]
IIb	C3, C1q	-	1–18 y	Frontal, temporal, occipital	[101]
Ia, IIa, IIb	FGF13	-	1–19 y	Frontal, temporal, parietal, and occipital	[40]
IIb	GRIN2A, GRIN2B, DLG4, DLG1, SLC17A7	-	1–47 y	Frontal, temporal	[102]
IIa, IIb, III	MMP1, MMP-9, MMP8, MMP2	-	2.7–35 y		[103]
IIa, IIb	FANCI, FANCA, BRCA2, RAD18, KEAP1	-	Not reported	Not reported	[104]
IIb	LAMP2, LAMP1, SQSTM1, ATG5	-	pediatric	Temporal neocortex	[105]
IHC, WB, and double-label immunofluorescence					
IIb	(DL-IF) SH3RF1, LILRB2, SHROOM3, ROCK1, ROCK2	-	1.7–12.5 y	Frontal, temporal, parietal, occipital	[106]
IIb	ADK	-	2.42–29 y	Frontal, temporal, occipital, parietal	[107]
IIa, IIb	NKCC1	NKCC2, GAD	3–54 y	Frontal, temporal, parietal, and occipital	[52]
IIb	DLG3, GRIN2B in postsynaptic density enriched fraction	-	14–40 y	mostly temporal	[108]
Ia, IIb	DCX	-	2–32 y	Frontal, temporal, occipital	[109]
IIb	TRPV1	-	1.5–9 y	Frontal, parietal, temporal, occipital cortex	[110]
IIb	-	BMP4	1.5–9 y	Frontal, parietal, temporal, occipital cortex	[111]
IIb	MMP-9	-	1.6–9.2 y	Frontal, parietal, temporal, occipital cortex	[112]
IIb	PDGFRB, CSPG4 (NG2), IBA1, GFAP	-	1–21 y	frontal, parietal, temporal	[113]
I, II	II, I: TBR1, OTX1, PAX6, MAP1B, II: ER81, NEFM	-	1–52 y	Temporal, Frontal	[80]
Ia, IIa, IIb	IL17RA, IL17A, TRAF3IP2, RELA	-	1–31 y	Frontal, temporal, parietal, occipital cortex	[114]
IIa, IIb	TLR4, IL1B, TNFA	-	1–13 y	Frontal, Temporal, Parietal	[42]
Mass Spectrometry and Other Methods (ISH, Enzyme-Linked Immunosorbent Assay (ELISA)					
IIb	TLR2, TLR4, AGER	-	11–26 y	Temporal, Frontal	[26]
IIa, IIb	NEFH	-	3–16 m	Frontal, Temporal, Parietal,	[49]
II	IL1B, IL6, CCL3, CCL4	IL-10	4–51 y	Frontal, Temporal	[24]
II	CNR1, RPS6	-	3–21 y	Frontal, Temporal	[29]
I, II	NKCC1 GABRA4, GABRA5, GABRG2	-	0–9 y and ≥10 y	Frontal, Temporal	[30]
IIa, IIb	pS6 present	-	1–42 y	Frontal lobe	[115]
IIb	C1QA/B/C, C3, AIF1, HLA-DRB1	-	2–57 y	temporal, frontal, parietal	[116]
CCD	64 proteins, including FSCN1, CRMP1, NDRG1, DPYSL5, MAP4, FABP3	89 proteins, including PRDX6 and PSAP	~7 y	neocortical tissue	[117]
IIa, IIb	SLC1A1	GLUL, SLC1A2	12–27 y	temporal, frontal, occipital	[118]
General	IL7	-	21 y	hippocampus	[119]
II	NES, CD34, GFAP, VIM, NEFL, GRIN1, GRIN2A, GRIA1, GRIA3, ABCB1	-	10–36 y	Frontal and temporal neocortex	[120]
II, IIIb	II, III: CD34 II: BCL2	-	3–51 y	Temporal neocortex	[121]

**Table 5:** Overlapping dysregulated proteins identified across studies. This table identified proteins in more than one study, categorized by their expression status (upregulated or downregulated) and associated FCD subtypes when available.

Publication							
[65]			+				
[78]					+		
[76]					+		
[69]		+		+			+
[43]					+		
[53]							
[60]		+					+
[73]		+					
[72]		+		+			+
[121]	+						
[29]					+		
[96]					+		
[115]					+		
[82]				+			
[52]						−	
[79]		+					+
[120]		+		+			+
[50]						−	
[99]					+		+
[71]		+					
[58]	+	+					
[86]						−	
[42]			+				
[113]		+					
[68]		+					
[98]						−	
[52]						−	
[83]	+			+	+		
[24]			+				
Protein	CD34	GFAP	IL1B	NES	RPS6	SLC12A5	VIM
Type		IIa	IIb	II	Mix		
Color							

“+”—increased expression. “−”—reduced expression.

**Table 6:** High-confidence consistently altered at both mRNA and protein levels across studies, with directionality and subtype specificity.

Overlaps	[93]	[29]	[37]	[32]	[40]	[30]	[105]	[35]	[106]	[114]	[88]	[33]	[47]	[34]	[44]	[38]	[20]	[24]
*FGF13*					+													
*TRPC1*														+				
*PANX1*			+															
*GPER1*				−														
*PRKACA*				−														
*CD47*												−						
*CD200*												−						
*SNCA*																−		
*NF-ΚB1*				+														
*PANX2*			+															
*ROCK1*									+									
*ROCK2*									+									
*IL17RA*										+								
*LAMP1*							+											
*LAMP2*							+											
*IL6R*	+																	
*HMGCS1*													+					
*HMGCR*													+					
*SQLE*													+					
*NEUROG2*																	+	
*RND2*																	+	
*CCL3*																		+
*CNR1*		+																
*GABRA5*						+												
*CD68*															+			
*IL2*								+										
*IL2RB*								+										
*IL2RG*								+										
*BDNF*											+							
*GABRD*							+											
Type					IIb				II					Mix				
Color																		

“+”—increased expression. “−”—reduced expression.

## Data Availability

This study does not generate original data. All the data analyzed in this study were obtained from previously published studies and are included in this manuscript or Supplementary Materials. The interactive tables and results are available in the Zenodo repository, accessible at https://doi.org/10.5281/zenodo.15786178 (accessed on 1 October 2025).

## Supplementary Materials

The following supporting information can be downloaded at: https://www.mdpi.com/article/10.3390/ijms26209909/s1. Reference [150] is cited in the Supplementary Materials.

## Abbreviations

FCD	Focal cortical dysplasia
CCD	Childhood cortical dysplasia
ILAE	International League Against Epilepsy
PRISMA	Preferred Reporting Items for Systematic Reviews and Meta-Analyses
PROSPERO	International Prospective Register of Systematic Reviews
iPSCs	Induced pluripotent stem cells
mTOR	Mechanistic Target of Rapamycin
PI3K	Phosphoinositide 3-kinase
Akt	Protein kinase B
NF-κB	Nuclear factor kappa B
RNA-Seq	RNA sequencing
PCR	Quantitative Polymerase Chain Reaction
RT-PCR	real-time quantitative polymerase chain reaction
WB	Western blot
IHC	Immunohistochemistry
LC–MS/MS	liquid chromatography–tandem mass spectrometry
MRI	magnetic resonance imaging
ELISA	Enzyme-Linked Immunosorbent Assay
ISH	In situ hybridization
DL-IF	Double-label immunofluorescence
iTRAQ	Multiplexed Isobaric Tagging Technology for Relative Quantitation
HGNC	HUGO Gene Nomenclature Committee
UniProtKB	Universal Protein Knowledgebase
gProfiler	Gene Enrichment Profiler
MOGHE	mild malformation of cortical development with oligodendroglial hyperplasia in epilepsy
LITT	laser interstitial thermal therapy
SEEG-RFTC	stereo-EEG-guided radiofrequency thermocoagulation
EEG	electroencephalography
