# Supplementary material for: Molecular Signature in Focal Cortical Dysplasia: A Systematic Review of RNA and Protein Data
Source: Int J Mol Sci. 2025 Oct 11;26(20):9909. doi: 10.3390/ijms26209909 (PMC12563006; doi:10.3390/ijms26209909)
Supplement: Supplementary file 1 [file ijms-26-09909-s001.zip › Supplementary S1.pdf]

PRISMA

PRISMA 2020 for Abstracts Checklist

Section and Topic	Item #	Checklist item	Reported (Yes/No)
**TITLE**			
Title	1	Molecular Signature in Focal Cortical Dysplasia: A Systematic Review of RNA and Protein Data	
**BACKGROUND**			
Objectives	2	Focal cortical dysplasia (FCD) is a major cause of drug-resistant epilepsy, but molecular mechanisms remain unclear. Numerous studies have analyzed RNA, protein, and microRNA alterations, yet findings often appear inconsistent across subtypes and methodologies. We conducted a systematic review to integrate transcriptomic, proteomic, and microRNA data from human FCD subtypes I–III and identify convergent molecular pathways.	
**METHODS**			
Eligibility criteria	3	We included peer-reviewed human studies reporting differentially expressed RNAs, proteins, or microRNAs in resected FCD tissue; reviews, case reports, and animal studies were excluded.	
Information sources	4	PubMed, Scopus, Web of Science, and Science direct were searched from inception to **August 2024**, and reference lists were screened manually.	
Risk of bias	5	Risk of bias was not formally assessed due to heterogeneity; limitations are discussed qualitatively	
Synthesis of results	6	Differentially expressed markers were categorized by FCD subtype and analyzed using pathway enrichment and network approaches.	
**RESULTS**			
Included studies	7	117 studies included	
Synthesis of results	8	Convergent dysregulation was observed in neuroinflammatory, synaptic, cytoskeletal, and metabolic pathways. Consistently altered genes (IL1B, TLR4, BDNF, HMGCR, ROCK2) and dysregulated microRNAs (hsa-miR-21-5p, hsa-miR-155-5p, hsa-miR-132-3p) were linked to PI3K–Akt–mTOR, Toll-like receptor, and GABAergic signaling. Overlapping transcript–protein patterns and subtype-specific molecular profiles were identified.	
**DISCUSSION**			
Limitations of evidence	9	Heterogeneity in classification systems, patient cohorts, and methodological approaches limited direct comparisons and prevented quantitative meta-analysis.	
Interpretation	10	This review provides the first cross-omics molecular framework of FCD, showing that convergent pathways unify heterogeneous findings and emphasize candidate biomarkers and therapeutic targets.	
**OTHER**			
Funding	11	This work was supported by the Ministry of Health of the Czech Republic in cooperation with the Czech	

PRISMA 2020 for Abstracts Checklist

Section and Topic	Item #	Checklist item	Reported (Yes/No)
		Health Research Council under project No NU21-04-00305 and project nr. LX22NPO5107(MEYS), Financed by the European Union-Next Generation EU.	
Registration	12	This review was registered with PROSPERO (registration number: CRD42024611156), and the protocol is available at http://www.crd.york.ac.uk/PROSPERO	

From: Page MJ, McKenzie JE, Bossuyt PM, Boutron I, Hoffmann TC, Mulrow CD, et al. The PRISMA 2020 statement: an updated guideline for reporting systematic reviews. BMJ 2021;372:n71. doi: 10.1136/bmj.n71. This work is licensed under CC BY 4.0. To view a copy of this license, visit https://creativecommons.org/licenses/by/4.0/

	Item #	Checklist item	Location where item is reported
**TITLE**			
Title	1	Molecular Signature in Focal Cortical Dysplasia: A Systematic Review of RNA and Protein Data	First page of manuscript
**ABSTRACT**			
Abstract	2	The abstract includes objectives, eligibility criteria, information sources (databases, last search), risk of bias note, methods of synthesis, key results (117 studies; convergent pathways; candidate markers), limitations, interpretation, funding, and registration (PROSPERO CRD42024611156). A completed PRISMA abstract checklist is provided in Supplementary Materials.	Abstract; PRISMA abstracts checklist supplied as Supplement
**INTRODUCTION**			
Rationale	3	Focal cortical dysplasia (FCD) is a major cause of drug-resistant epilepsy, but molecular studies at RNA, protein, and microRNA levels have produced inconsistent and fragmented results. Previous reviews typically considered single omics layers, limiting identification of convergent mechanisms. This review addresses that gap by systematically integrating transcriptomic, proteomic, and microRNA data to unify heterogeneous findings, clarify subtype-specific alterations, and provide a framework for biomarker discovery and therapeutic development.	Introduction
Objectives	4	To systematically integrate transcriptomic, proteomic, and microRNA data from human FCD subtypes (I–III) in order to identify convergent molecular pathways, characterize subtype-specific mechanisms, and emphasize candidate biomarkers and therapeutic targets.	End of Introduction
**METHODS**			
Eligibility criteria	5	peer-reviewed studies on human FCD tissue reporting differentially expressed RNAs, proteins, or miRNAs (2004–August 2024, English). Excluded: animal-only studies; genetic mutation studies not reporting expression; reviews, case reports, letters, conference abstracts; studies without molecular data; morphology-only or non-English full texts. Data grouped by omics type and FCD subtype.	Method 2.2 Eligibility criteria.
Information sources	6	We searched PubMed, Web of Science, Scopus, and ScienceDirect. Reference lists of included studies were also screened. The last database search was conducted in August 2024.	Method 2.1 Search Design
Search strategy	7	Search items included: (Focal Cortical Dysplasia OR FCD OR Cortical Dysplasia) AND (miRNA OR microRNA OR miR), (Focal Cortical Dysplasia OR FCD OR Cortical Dysplasia) AND (Transcriptomics OR Microarray OR RNA-sequencing), (Focal Cortical Dysplasia OR FCD OR Cortical dysplasia) AND (Proteomics OR protein profiling OR Mass spectrometry) AND (Focal Cortical Dysplasia OR FCD OR Cortical dysplasia) AND (protein expression OR protein analysis OR western blot OR ELISA OR immunohistochemistry OR IHC OR immunostaining. Reference lists of relevant articles were also screened manually to identify additional eligible studies. The complete screening process is illustrated in Figure 1.	Method 2.1 Search Design

	Item #	Checklist item	Location where item is reported
Selection process	8	Two reviewers independently screened titles, abstracts, and full texts. Disagreements were resolved by discussion with a third reviewer. Numbers of included and excluded records are reported in the PRISMA flow diagram (Figure 1).	Method 2.3 Study Selection workflow; Figure 1 (PRISMA)
Data collection process	9	Two reviewers independently extracted data using a standardized form. Discrepancies were resolved by consensus or third-party adjudication. Extracted items included study characteristics, patient demographics, methodology, molecular findings, and statistical significance.	Method 2.4 Data Extraction
Data items	10a	The primary outcomes were differentially expressed molecular entities in human FCD tissue, including miRNAs, mRNAs, and proteins. For each, we recorded direction of dysregulation (upregulated or downregulated), statistical significance (p-values, fold changes where available), and functional annotation. Molecular outcomes were subsequently mapped to biological pathways and integrated into cross-omics networks.	Method 2.4 and 2.5
	10b	Study-level and contextual variables were collected: FCD subtype (I–III), sample type (surgical vs. control), number of cases and controls, patient age, lesion localization, and disease duration (where reported). Methodological details included molecular platform (RNA-seq, microarray, RT-qPCR, LC-MS/MS, IHC, Western blot), and validation methods. Gene and protein identifiers were standardized using HGNC, UniProtKB, and GeneCards.	Method 2.4 and 2.5
Study risk of bias assessment	11	No formal risk-of-bias tool was applied due to methodological heterogeneity across molecular studies. Instead, potential sources of bias such as classification criteria, sample quality, and assay differences were qualitatively evaluated and discussed in Section 5.1 (Biological and Methodological Heterogeneity).	2.5
Effect measures	12	Extracted measures included fold change, log2 fold change, and p-values when available; presence/absence data from IHC/WB were recorded qualitatively. No pooled quantitative effect sizes were calculated; synthesis was descriptive.	2.5
Synthesis methods	13a	For each synthesis, we grouped studies by both molecular layer (microRNA, RNA transcript, protein) and FCD subtype (I–III). Studies were eligible for inclusion if they provided original human tissue data with molecular findings. Gene, protein, and miRNA identifiers were standardized using HGNC, UniProtKB, and GeneCards to allow comparability across datasets.	Method 2.4 and 2.5
	13b	Before analysis, we harmonized identifiers, reconciled synonyms. For studies reporting only presence/absence (IHC/Western blot), these findings were included qualitatively. Overlapping datasets were checked to avoid duplication.	Method 2.5
	13c	Results were presented using structured summary tables and visualizations. Tables detail dysregulated molecules, subtype associations, methods, and references. Figures include Sankey diagrams linking molecules to pathways, Circos plots, and cross-omics networks (built with tidygraph, ggraph, and ggplot2).	Method 2.5
	13d	Qualitative synthesis was chosen due to methodological and biological heterogeneity. Convergence was defined as recurrent dysregulation of molecules and pathways across independent studies within and across omics layers. Pathway enrichment was performed with gprofiler2 and clusterProfiler (GO BP, WikiPathways) to contextualize signals.	2.5
	13e	We explored heterogeneity qualitatively. Key sources included classification systems (Palmini vs. ILAE), patient age, lesion localization, disease duration, tissue acquisition (surgical vs. postmortem), cell type composition (balloon cells, gliosis), and platform differences (RNA-seq, microarray, RT-qPCR; LC-MS/MS, 2D-DIGE, IHC, Western blot). These	5.1

	Item #	Checklist item	Location where item is reported
		factors were highlighted in Section 5.1.	
	13f	We did not perform formal sensitivity analyses because no quantitative synthesis was undertaken. Instead, robustness was assessed qualitatively by noting consistent findings across independent cohorts and methodological platforms.	2.5, 5.1
Reporting bias assessment	14	No formal statistical assessment of reporting bias was conducted. Potential selective reporting and publication bias are acknowledged in Section 5.1.	5.1
Certainty assessment	15	Certainty of evidence was not formally graded. Instead, findings consistently observed across multiple independent studies and omics layers were interpreted as higher-confidence signals.	2.5
**RESULTS**			
Study selection	16a	Out of 7088 retrieved records, 5697 unique records remained after deduplication. After title/abstract screening, 217 full-texts were assessed, and 117 studies met inclusion criteria. The PRISMA flow diagram is provided in Figure 1.	2.3; Figure 1 (PRISMA).
	16b	Full-text exclusions: non-human (n=8), mutation-only (n=14), incomplete or non-specific molecular data (n=72), morphology-only (n=2), non-English full text (n=2), letters (n=2).	2.3
Study characteristics	17	The 117 included studies comprised miRNA (n=8), transcriptomic (n=28), and proteomic (n=98) analyses (some overlapping). All used resected human FCD tissue with various controls. Platforms included RNA-seq, microarrays, RT-qPCR, LC-MS/MS, 2D-DIGE, IHC, and Western blotting. Subtype distribution was uneven, with type IIb most frequently studied.	Result3 and Supplementary tables
Risk of bias in studies	18	No formal tool was applied. Methodological and biological limitations (classification systems, tissue quality, and platform variability) are discussed in Section 5.1.	5.1
Results of individual studies	19	For each included study, we reported the following: study characteristics (FCD subtype, lesion site, and method), molecular findings (differentially expressed transcripts, proteins, or microRNAs, including the direction of change), and subtype-specific results. Data are presented in detailed supplementary tables (organized by omics type and subtype) with references to original studies. In the main text, consistently dysregulated targets (e.g., IL1B, TLR4, BDNF, HMGCR, ROCK2) are emphasized.	Results sections (targets and pathways); Supplement/Zenodo tables referenced in Data Availability.
Results of syntheses	20a	We conducted qualitative syntheses by omics layer (miRNA, RNA transcript, protein) and by FCD subtype (I–III). Contributing studies varied in classification era (Palmini vs. ILAE), patient age (pediatric/adult), lesion site, sample quality and and platforms (RNA-seq/microarray/RT-qPCR; LC-MS/MS/2D-DIGE/IHC/WB). No formal risk-of-bias tool was applied due to methodological heterogeneity; potential biases are considered qualitatively. Convergent signals across independent studies highlighted neuroinflammatory, synaptic, cytoskeletal, and metabolic pathways.	Discussion 4.1–4.2; 5.1 (heterogeneity).
	20b	Not applicable. No quantitative meta-analysis or statistical pooling was performed because of heterogeneity in study designs, measurements, and reporting.	Methods2.5; Discussion5.1.
	20c	Heterogeneity was explored qualitatively by stratifying findings across subtypes (I–III), omics layers, and methodologies. Differences were frequently attributable to classification systems (Palmini vs. ILAE), cohort composition (age, lesion localization, disease duration), tissue quality/cell-type composition (gliosis/balloon cells), and	5.1

	Item #	Checklist item	Location where item is reported
		platform/threshold choices. Despite these sources of variation, recurrent pathway-level convergence (e.g., TLR/IL signaling, PI3K–Akt–mTOR, GABAergic signaling, cholesterol/lipid biosynthesis) persisted across multiple independent datasets.	
	20d	Not performed. No quantitative synthesis was undertaken; instead, robustness was judged qualitatively by recurrence across independent cohorts and across omics layers/platforms.	Methods 2.5; Discussion 5.1
Reporting biases	21	Not assessed formally. Potential publication/selective reporting biases are acknowledged given the diversity of platforms and reporting thresholds; we mitigated this by screening reference lists and including non-significant/negative findings when reported. Limitations related to reporting bias are discussed qualitatively.	2.5, 5.1
Certainty of evidence	22	Not graded using a formal tool. We interpreted signals as higher-confidence when (i) replicated across ≥1 independent study within an omics layer, and/or (ii) observed across multiple omics layers (transcript–protein agreement), with consistent directionality and pathway assignment. Single-study or single-platform findings were interpreted cautiously.	Methods 2.5 (short statement); Discussion 4.1–4.2, 4.6, 5.1.
**DISCUSSION**			
Discussion	23a	Cross-omics integration indicates convergent dysregulation of neuroinflammatory, synaptic, cytoskeletal, and metabolic pathways in FCD, linking TLR/IL signaling and PI3K–Akt–mTOR activity to GABAergic dysfunction and lipid/cholesterol biosynthesis. These findings reconcile previously fragmented molecular observations and align with recent single-cell/spatial data that localize programs to specific neuronal, glial, and vascular compartments.	Discussion 4.1–4.2, 4.6–4.9
	23b	Evidence is limited by changes in classification (Palmini vs. ILAE), uneven subtype representation (particularly greater evidence for IIb), cohort variability, tissue quality/cell-mix differences, and platform-specific detection/threshold choices.	Discussion5.1
	23c	No formal risk-of-bias or certainty grading, no reporting-bias analysis, and no quantitative meta-analysis/sensitivity analyses were conducted due to heterogeneity; synthesis relied on qualitative convergence and enrichment-based integration.	Discussion 5.1; Methods 2.5
	23d	Findings prioritize candidate biomarkers (circulating miRNAs such as hsa-miR-21-5p, hsa-miR-132-3p) and therapeutic targets (IL1B/TLR4; cholesterol biosynthesis enzymes; ROCK2). Standardized, multicenter, multi-omic studies with cell-type/spatial resolution and consistent reporting are recommended to support precision-medicine strategies in FCD.	Discussion 4.7; Conclusions 6; Future Recommendations 7.
**OTHER INFORMATION**			
Registration and protocol	24a	Registered in PROSPERO: CRD42024611156.	Methods 2 (Registration statement)
	24b	protocol is available at http://www.crd.york.ac.uk/PROSPERO	Methods 2
	24c	No amendments were made to the registered protocol.	Method 2

PRISMA

PRISMA 2020 Checklist

	Item #	Checklist item	Location where item is reported
Support	25	This work was supported by the Ministry of Health of the Czech Republic in cooperation with the Czech Health Research Council under project No NU21-04-00305 and project nr. LX22NPO5107(MEYS), Financed by the European Union-Next Generation EU.	Funding
Competing interests	26	The authors declare no conflict of interest.	Conflicts of Interest
Availability of data, code and other materials	27	Data supporting the review (extraction tables/figure source data) are available at Zenodo (DOI: 10.5281/zenodo.15786178). Interactive tables are provided in the Supplementary Materials. Analysis scripts are available from the authors upon reasonable request.	Data Availability section; Supplementary Materials

From: Page MJ, McKenzie JE, Bossuyt PM, Boutron I, Hoffmann TC, Mulrow CD, et al. The PRISMA 2020 statement: an updated guideline for reporting systematic reviews. BMJ 2021;372:n71. doi: 10.1136/bmj.n71. This work is licensed under CC BY 4.0. To view a copy of this license, visit https://creativecommons.org/licenses/by/4.0/
