# Supplementary material for: Molecular Signature in Focal Cortical Dysplasia: A Systematic Review of RNA and Protein Data
Source: Int J Mol Sci. 2025 Oct 11;26(20):9909. doi: 10.3390/ijms26209909 (PMC12563006; doi:10.3390/ijms26209909)

Supplementary Figure: Schematic overview of the computational pipeline used for integrating protein, RNA, and microRNA (miRNA) data in FCD-related studies. Data were filtered based on gene/protein names, expression status, and FCD subtype using dplyr and tidyR. Gene, protein, and miRNA names were unified using standardized identifiers (Entrez ID, HGNC, UniProt, RefSeq, and MultiMiR). Interactive tables were generated using the DT package. Enrichment analyses were conducted using enrichR and gprofiler, and RNA–miRNA interactions were examined via MultiMiR and EnrichR. Final results were visualized using bar plots, dot plots, circular plots, and Sankey diagrams.

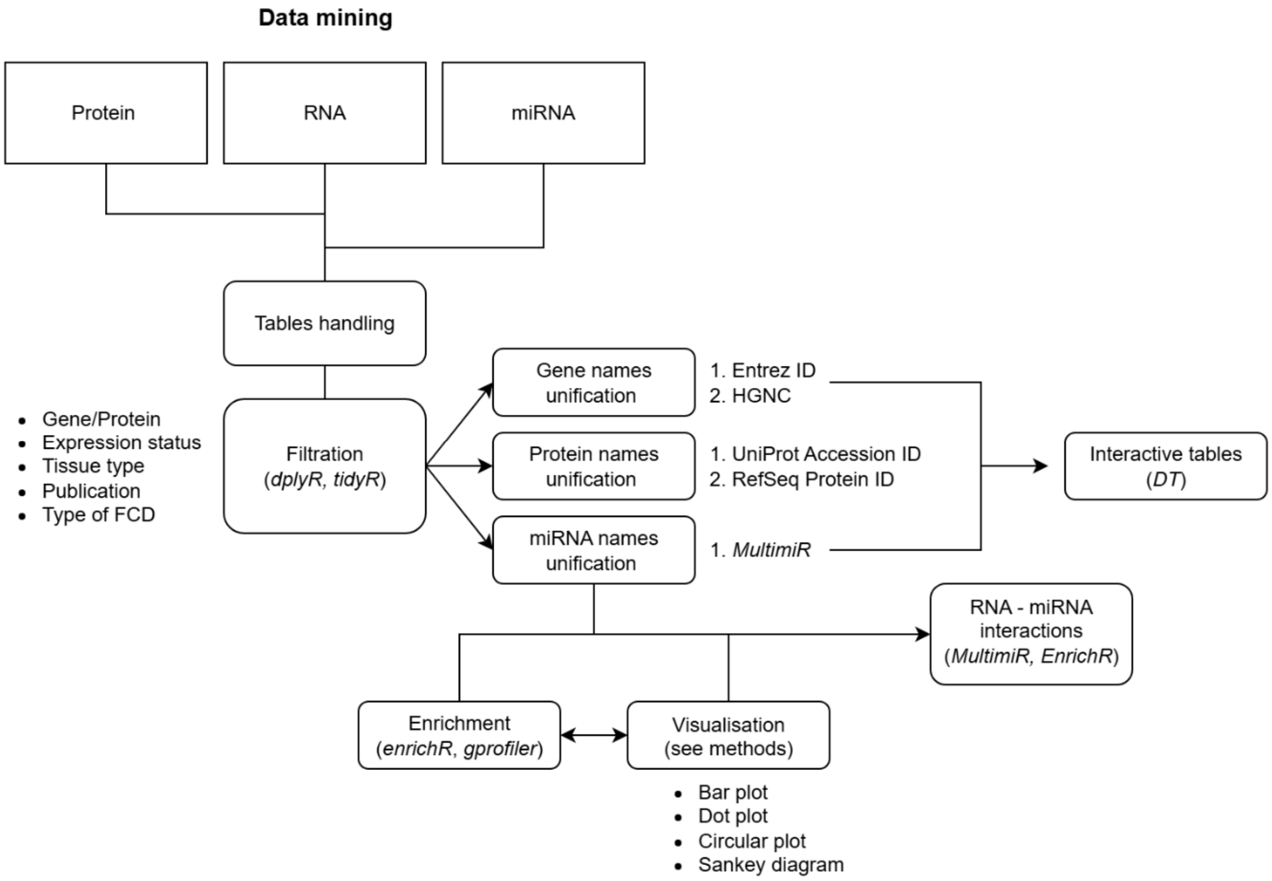

Supplement: Supplementary file 1 [file ijms-26-09909-s001.zip › Supplementary S2.pdf]
